# Information exchange in laboratory markets: competition, transfer costs, and the emergence of reputation

**DOI:** 10.1007/s10683-020-09652-0

**Published:** 2020-04-10

**Authors:** Roman Hoffmann, Bernhard Kittel, Mattias Larsen

**Affiliations:** 1 Wittgenstein Centre for Demography, Global Human Capital (Univ. Vienna, IIASA, VID/ÖAW), Vienna Institute of Demography/Austrian Academy of Sciences, Vienna, Austria; 2 Potsdam Institute for Climate Impact Research, Potsdam, Germany; 3 Department of Economic Sociology, University of Vienna, Vienna, Austria; 4 Department of Agri-Business, United Nations Industrial Development Organization (UNIDO), Vienna, Austria

**Keywords:** Reputation, Trust, Moral hazard, Information sharing, Competition, Experiment, C9, D6, D8, G1, L1

## Abstract

Public reputation mechanisms are an effective means to limit opportunistic behavior in markets suffering from moral hazard problems. While previous research was mostly concerned with the influence of exogenous feedback mechanisms, this study considers the endogenous emergence of reputation through deliberate information sharing among actors and the role of barriers in hindering information exchange. Using a repeated investment game, we analyze the effects of competition and transfer costs on players’ willingness to share information with each other. While transfer costs are a direct cost of the information exchange, competition costs represent an indirect cost that arises when the transfer of valuable information to competitors comes at the loss of a competitive advantage. We show that barriers to information exchange not only affect the behavior of the senders of information, but also affect the ones about whom the information is shared. While the possibility of sharing information about others significantly improves trust and market efficiency, both competition and direct transfer costs diminish the positive effect by substantially reducing the level of information exchange. Players about whom the information is shared anticipate and react to the changes in the costs by behaving more or less cooperatively. For reputation building, an environment is needed that fosters the sharing of information. Reciprocity is key to understanding information exchange. Even when it is costly, information sharing is used as a way to sanction others.

**Electronic supplementary material:**

The online version of this article (10.1007/s10683-020-09652-0) contains supplementary material, which is available to authorized users.

## Introduction

Imperfect information and insufficient contractual enforcement can undermine trust and cause large inefficiencies in markets that suffer from moral hazard problems (Akerlof 1970). Reputation mechanisms, i.e. the availability of information about the past conduct of market participants, can serve as an effective remedy to overcome trust problems. Learning about the past behavior of (potential) transaction partners helps reduce uncertainties and creates incentives to cooperate in order to avoid exclusion from future transactions (Anderhub et al. 2002; Huck et al. 2012).

While there is strong evidence that reputational information fosters trust and limits opportunistic behavior in markets, only a few studies have analyzed the conditions under which reputation and feedback mechanisms emerge (Abraham et al. 2016; Gërxhani et al. 2013; Jappelli and Pagano 1999). The automatic provision of feedback, for example, in online markets or through third party institutions such as credit bureaus and consumer protection agencies, can facilitate information exchange and help overcome barriers to information sharing among market participants. In other cases, the sharing of information does not occur automatically, but requires the strategic decisions of actors who weigh the benefits of information sharing against its costs (Pagano and Jappelli 1993; Frey 2017). This creates a *second*-*order social dilemma* (Fehr and Gächter 2002; Ostrom 1990) in which individuals may decide not to share their experiences, even though the transfer of information helps to establish market transparency, to bridge information asymmetries, and to overcome trust and moral hazard problems.

Microfinance is an example of a market suffering from informational asymmetries for which—despite recent attempts to establish stronger external regulation—there are no institutional solutions to facilitate the sharing of information (Cason et al. 2012; de Janvry et al. 2010; Giné et al. 2012; Luoto et al. 2007). The resulting informational deficit and low information exchange, which are often due to fierce competition among the microfinance providers, can generate harmful effects for clients, who experience high interest rates or over-indebtedness due to lending from multiple sources. They can also be harmful for the microfinance organizations, who have to deal with adverse selection effects and hard to predict default risks. If information about borrowers is shared, this typically occurs in an informal way, for example, by word of mouth. Other examples include employers informally exchanging information about job candidates (Gërxhani et al. 2013) or purchasing companies about suppliers (Buskens and Raub 2002).

Focusing on markets without institutionalized feedback mechanisms, our study (1) analyzes the conditions under which information is voluntarily shared among actors, (2) documents the consequences of a lack of information exchange and information asymmetries, and (3) highlights settings that could benefit from external regulation. We study the endogenous sharing of information in a laboratory experiment using repeated investment games to replicate markets suffering from informational asymmetries and moral hazard (Berg et al. 1995). The game is played between a first mover, the *trustor*, and a second mover, the *trustee*. Due to the sequential structure of the game, the trustor’s payoff after engaging in a transaction will depend on the cooperation of the trustee.

In our design, trustors can decide whether or not to share information about previous transactions with others. The sharing of information may come at a cost. Conceptually, we distinguish between direct and indirect costs. Direct costs refer to costs that arise directly in the information transfer process, such as efforts to inform others, which are randomly varied in a within-subject treatment. Indirect costs, on the other hand, arise as a consequence of one-sided *competition* between market participants, the group of trustors in our case. With a rivalry in payoffs, information about the quality of trustees has a private value for trustors and concealing it may create a competitive advantage.

Our study contributes to the literatures on strategic information sharing and the role of exchange barriers and costs for the functioning of reputation mechanisms (Brown and Zehnder 2010; Frey 2017; Gërxhani et al. 2013; Abraham et al. 2016). We consider the effects of voluntary information sharing and of exchange barriers on both trustors and trustees. Our results show that barriers to information exchange in the form of direct transfer costs and competition affect both types of players, leading to interesting dynamics. While free information exchange significantly improves trust and welfare, both types of costs diminish information sharing among trustors, making it less valuable for trustees to build a good reputation. We find evidence for a non-linear cost elasticity function, with even small direct costs leading to a disproportionally large drop in information sharing. The one-sided competition among trustors has a dual effect on the market: While it leads to a substantial decrease in information sharing and less trustworthiness, it encourages trustors to take more risks and to make higher investments to outperform competitors, which absorbs some of the negative welfare effects of reduced information sharing. Finally, there is strong evidence that the amount of information sharing by trustors is influenced by reciprocal motives towards fellow trustors and trustees alike.

In our design, we abstract from many features that characterize markets suffering from moral hazard. Most notably, our design does not include a pricing mechanism and restricts free choice of trading partners. While these are important features of real markets, our stylized laboratory design focuses on the core issues of interest for our study, i.e. the role of voluntary information sharing and the consequences of different exchange barriers on trust and reputation building. The findings are relevant for markets where reputational information is important but no institutionalized information sharing or feedback mechanisms exists. Even if there is a common interest in exchanging information, transfer costs and competition may diminish information exchange, with major implications for the entire market.

The remainder of the paper is structured as follows. Section 2 discusses the existing theoretical and empirical literature. Section 3 introduces the experimental design and the measurement of key variables. Section 4 presents the results and Sect. 5 discusses the findings and conclusions. The main text is accompanied by supplementary material offering a more detailed overview of the literature (S1), additional descriptive statistics (S2), model variations and sensitivity checks (S3), and more information on the experimental procedures and instructions (S4–S7).

## Information sharing and reputation

Players have an incentive to build a reputation for trustworthiness when others can observe them or are informed about their behavior and when future transactions are valuable and sufficiently likely to occur (Schelling 1960; Kreps and Wilson 1982). Reputation mechanisms, i.e. the institutionalized provision of information about market actors, have been shown to have large social benefits, for instance, in credit markets (Millon and Thakor 1985; Brown and Zehnder 2005; Padilla and Pagano 2000) or in online trading (Bolton et al. 2005; Resnick et al. 2006; Houser and Wooders 2006; Resnick and Zeckhauser 2002; Bolton et al. 2004; Fehr et al. 2018; Bolton et al. 2013). Laboratory experiments also suggest that mechanisms for building good reputations positively influence trust and efficiency in markets prone to moral hazard (Huck et al. 2012; Keser 2002; Bolton et al. 2004; Bohnet and Huck 2004; Bohnet et al. 2005).

In situations where no institutionalized feedback mechanisms exist, the sharing of information relies upon the strategic decisions of agents who weigh the benefits of sharing information, such as receiving information from others in the future, against its costs. While previous studies have shown that actors are willing to share information with each other when this is free of costs, little is known about how barriers to information exchange affect the behavior of both trustors and trustees and the dynamics in the market. Following Pagano and Jappelli (1993) and Padilla and Pagano (1997), we distinguish between direct and indirect costs of information exchange.

*Direct costs* refer to all costs that occur in the information transfer process. For instance, the sharing of information about a deviant trustee requires that a trustor seeks contact with others and shoulders additional administrative work, which may reduce incentives to share information. Gërxhani et al. (2013) analyze the effect of direct transfer costs in employer networks in the laboratory, in which employers as trustors can learn the quality of applicants by sharing information with each other. They initially find a high level of information sharing, but once transfer costs are introduced, sharing declines substantially. Similarly, Abraham et al. (2016) find that information transmission is most likely to occur if it is costless. Even with small costs, sharing decreases to a very low level, suggesting a high cost elasticity.

In addition to direct costs, information sharing may also entail *indirect costs*, which are costs that arise due to the transfer of valuable information to competing economic agents without obtaining direct benefits in return (Pagano and Jappelli 1993). If information has a private value, sharing it with competitors may help the latter to compete more aggressively. Trustors hence have to weigh the benefits of a functioning reputation system against the loss of one’s information advantage, as in the case of the microfinance markets described above. Essentially, sharing information takes the form of a second order public good game, which creates a social benefit but may not be individually rational (Fehr and Gächter 2002). The trustors face an *information sharing dilemma*: Should they share information with others in order to help establish a reputation mechanism even if this is costly for them?

While competition among trustees has been shown to significantly raise trustworthiness and overall efficiency in trust games (Huck et al. 2012; Brown et al. 2004; Cabrales et al. 2011), there is little evidence about its impact on informational exchanges and the establishment of reputation mechanisms. Brown and Zehnder (2010) analyze voluntary information sharing in a laboratory credit market and find that competition significantly reduces the willingness to share experiences (see also Brown and Zehnder 2007). With increasing informational asymmetries, the negative effect of competition on information sharing decreases, suggesting that lenders recognize the need for a reputation mechanism when there are strong incentives for borrowers to defect (see also Banerjee et al. (2012) for a field experimental study on the diffusion of information about rival goods).

Previous research has mainly been concerned with the impact of information exchange barriers on trustors’ willingness to share information with each other. Our study contributes to the literature by studying the effects on both trustors’ and trustees’ behavior. While the exchange barriers affect only the former directly, the latter might also react to the changes. Fearing less information sharing and sanctioning, trustees might decide to behave less cooperatively. In our design, we consider the effects of both direct transfer costs and competition on information sharing and trust in the game. In addition, we study the mechanisms underlying the observed behavioral dynamics. In particular, we explore the role of reciprocal motives (Fehr and Gächter 1998; Falk and Fischbacher 2006), which have previously been shown to be of relevance for feedback giving (Bolton et al. 2011, 2013; Diekmann et al. 2014) and voluntary information exchange in markets (Abraham et al. 2016).

## Experimental design and measurement

### The investment game

**Fig. 1:**
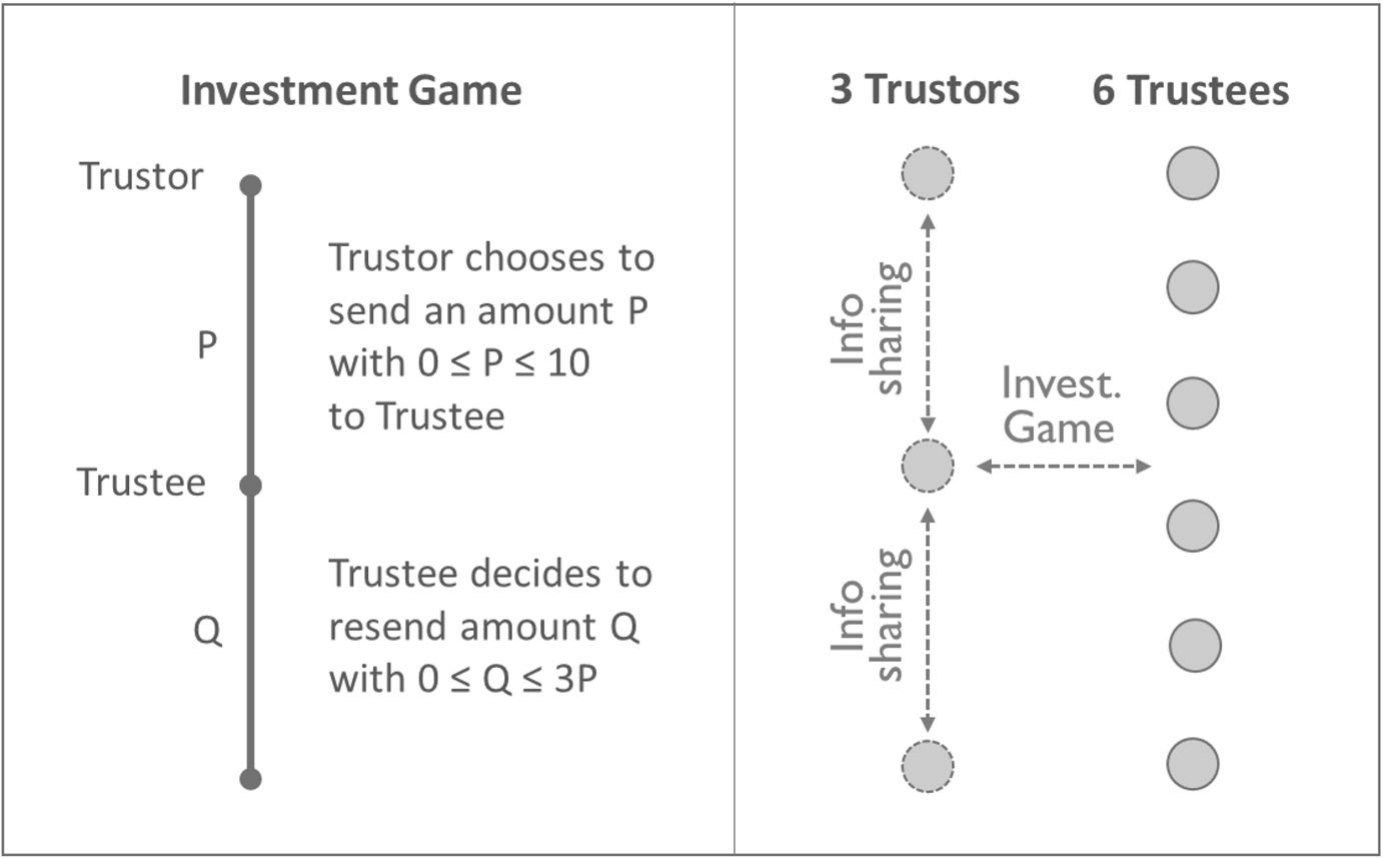
Investment game and market structure

We consider a repeated investment game with two player types, trustors and trustees, as depicted in Fig. 1 (Berg et al. 1995). The investment game resembles the standard trust game in its sequential structure. All trustors receive an initial endowment of 10 experimental tokens. In the first stage of the game they can send an amount P (the investment) out of their endowment (0 ≤ P ≤ 10) to the matched trustee. If the trustor decides to send nothing, the interaction ends (equivalent to the outside option in the trust game). If the trustor sends P > 0, this amount will be tripled and the trustee receives 3P. In the next stage of the game the trustee decides how many tokens Q she sends back to the trustor. This amount Q ranges from zero to a maximum of three times the sent amount P and can take integer values (0 ≤ Q ≤ 3P).

The trustor’s and the trustee’s payoffs are 10 – P + Q and 3P – Q, respectively. By choosing Q, trustees decide whether they want to reward trust or to defect. We speak of defection or betrayal if Q < P. In this case, it would have been better for the trustor to choose the outside option and not send P.

In the one-shot game, the payoff-maximizing behavior for the trustee is to send nothing back to the trustor. Anticipating the trustee’s defection, the trustor should not send anything in the first place. If the game is repeated for several rounds, trustors can learn about the trustees, which may create incentives for them to build a reputation of being trustworthy and thus foster cooperation (Schelling 1960; Kreps and Wilson 1982).

### The market structure

We analyze an experimental market consisting of nine participants, three trustors (named A-players) and six trustees (named B-players), as illustrated in Fig. 1. At the beginning, each player receives a unique trustor or trustee ID (A1–A3, B1–B6). Both the IDs and the composition of the groups remain constant throughout the game. The experiment lasts for at least 24 rounds. After the 24th round it ends with probability 0.5 in every consecutive round. Each of the identical rounds consists of three stages. Note that we are not only interested in the interactions between trustors and trustees, but also in the relationships among trustors. The instructions of our experiment are displayed in the supplementary material (S4).

- *Stage 1* In every round, each of the three trustors is randomly matched with one trustee. In the beginning of the round, both players learn the identity of their counterpart. The three trustees who were not matched with a trustor pause for one round. Instead of participating in the investment game, they can solve up to six arithmetic tasks and earn an additional income to compensate for the lost income during their pause (equivalent payoffs compared to players in the game). For each correctly solved task they can earn three additional experimental tokens.
- *Stage 2* In the second part, the matched trustors and trustees play the investment game as described above.
- *Stage 3* If P > 0 is sent, the trustor obtains a message about Q, the amount sent back by the matched trustee. All players are informed about their payoffs for this round as well as their cumulated payoffs from all preceding rounds.

We use a larger number of trustees than trustors to reduce the probability of players interacting too often during the game and to give trustors an additional incentive to share information in order to establish a public reputation system. We chose this design feature because it allowed us to ensure the identifiability of the interaction partners and to increase the size of the considered market without making the game too complex for the participants. Even though players interacted on average only 4 times in each session (~ 16.7% of all rounds), our chosen design might have given rise to direct reciprocity and private learning, possibly reducing the impact of the public reputation mechanism (Fréchette 2012). While this should not directly affect the estimation of the treatment effects, we tested for the robustness of our main findings in multiple sensitivity checks accounting for direct reciprocity and other features of our design (see supplement S3). In addition, in our main models, we control for the number of previous interactions between a trustor and a trustee to capture, at least in part, potential direct reciprocity effects.

### Treatments

Table 1 gives an overview of the treatment conditions considered in this study and the sample size in each treatment arm. In the baseline treatment (*base*), as described above, trustors have no possibility of sharing information with each other. They can collect private information about their past interaction partners, but they cannot share their experiences with others.

**Table 1:** Experimental treatments

	Indirect costs		Direct costs
	No competition	Competition	Varying transfer costs [0–1]
No information sharing	*Baseline (base)* 1080 Observations 45 Subjects 5 Groups	*Competition (com)* 1080 Observations 45 Subjects 5 Groups	
Information sharing	*Info sharing (inf)* 1089 Observations 45 Subjects 5 Groups	*Info sharing*—*competition (inf*-*com)* 1089 Observations 45 Subjects 5 Groups	*Direct costs* Within-subject treatment Only if sharing is possible

In the *information sharing treatment (inf)*, trustors were allowed to share their experiences with other trustors after playing the investment game. They could share information with none, one, or both of the other trustors. If information was shared, the receiving trustor was informed in the next round about the ID of the sending trustor, the ID of that trustor’s transaction partner in the previous round, the amount sent P, the tripled amount 3P, and the returned amount Q. Trustors could also send information if they sent nothing to their interacting trustee. In this case, other trustors were informed that the respective trustor has decided not to interact with the particular trustee in this round. All details were provided in the form of a history table displaying the experiences from all previous rounds. Note that the establishment of a reputation mechanism was truly endogenous here as it depended only on the decisions of the trustors. This situation most closely resembles a market, where information sharing is not mandatory and does not occur in an automatic way. The trustees were not informed about whether information was shared about them or not. We measured their beliefs in order to understand how their expectations influenced their decision-making.

In the *competition treatment (com)*, a trustor’s final payoff not only depended on the behavior of the exchange partner, but also on the overall performance of the other trustors (negative complementarity or rivalry in payoffs). We used a tournament mechanism to induce competition among the players. Tournament schemes have been used in other studies, for instance on the effects of competition on performance and cheating (Schwieren and Weichselbaumer 2010) and on differential behavioral reactions to competition (Niederle and Vesterlund 2007; Charness and Villeval 2009). At the end of each round, together with the payoffs, trustors were shown a ranking based on their accumulated payoffs from the previous rounds. Players’ positions in the ranking depended on their success in interacting with trustees in the previous rounds and hence on their ability to screen and to identify good and bad trustees. Every eighth round, the first and second ranked trustors received a substantial additional payoff of 10 € and 5 €. After each eighth round, the count of accumulated payoffs was set to zero and the ranking restarted. This ensured that competition remained vibrant and that players did not give up competing just because they felt that they could not catch up with the others. It also allowed us to allocate a sufficiently high bonus payoff to the best performing trustors, which was salient to the players.

In the *sharing*-*competition treatment (inf*-*com)*, the competing trustors had the possibility of sharing information with each other after learning about their position in the ranking. Compared to the simple information sharing treatment, the private value of information increased for the trustors, because the shared information could potentially benefit the competitor in their screening and selection of appropriate interaction partners. These indirect costs are expected to diminish information sharing compared to the simple information sharing treatment.

Next to these between-subject treatments, we included an additional *within*-*subject treatment* in which we varied the direct transfer costs for sharing information. In the sharing treatments, both trustors and trustees were informed in the first stage of the game about the direct costs of information transfers to other trustors in this round. In each round, costs were drawn randomly from a uniform distribution between 0 and 1 in increments of 0.1, which made it possible to analyze nonlinearities in the cost elasticities.^1^

A limitation of the tournament mechanism was that it not only induced competition in payoffs, but also led to an increase in the total payoffs of the trustors. This might have resulted in an income effect, which cannot be perfectly distinguished in our design from the theoretically relevant competition effect. To account for this issue, we controlled for the accumulated payoffs of all players and the receipt of a bonus payment by trustors in the models. None of these variables was found to significantly influence trustors’ behavior in the game and their willingness to engage in the competition. In addition, in the questionnaire at the end of our experiment, 84% of trustors confirmed that they perceived other trustors as competitors because of the ranking, suggesting that competition among the players was successfully induced. To test for the robustness of our other treatments (information sharing and direct costs), we present additional models in the supplementary material, which separate the data analysis for competition and non-competition treatments (S3.3).

### Measurement and procedures

Several indicators were used to measure the effects of the treatment conditions on the main outcomes of the game, namely information sharing, trustworthiness, willingness to trust, and welfare. Trustors could *share information* with none, one, or both of the other trustors. Most information sharing occurred with both other trustors (only 3.6% of all information exchanges were bilateral). Consequently, we captured the trustor’s willingness to share information with a dummy variable (I) taking the value 1 if a trustor shared information with others in the respective period. Naturally, this outcome could only be analyzed in treatments in which information sharing was in principle possible, i.e. information sharing with and without competition.

*Trustworthiness* is the trustee’s willingness to reward trust, which is reflected in the amount Q returned to the trustor. It is captured in the form of a return on investment (ROI), i.e. the ratio of the returned amount to the sent amount Q/P. The resulting variable can take on values from 0% (nothing was returned) to 300% (the entire tripled amount was returned). The trustworthiness indicators could only be calculated if the trustor sent at least a small amount (P > 0) in the respective period.

A trustor’s *willingness to trust* the interaction partner was assessed with the continuous sent amount P [0–10]. The generated welfare was measured separately for the trustors and trustees and is based on the payoffs from the game in each round, not considering bonuses or payoffs derived from the arithmetic calculations.

Information about *players’ beliefs* was collected in all stages of the game. After sending P > 0, trustors were asked for the amount they believed their matched trustee would send back. In the information sharing treatments, trustees were asked about whether or not they believed that information was shared about them in the respective round. These variables allowed us to determine whether players’ expectations explained their behavior in the game. All belief measurements were incentivized: Trustors received one additional token for correctly estimating the amount sent back by their trustees (up to an interval of ± 1 token). Trustees received two extra tokens for correct beliefs about the trustors’ information sharing decisions.

The experiments were conducted between June 2015 and February 2016 at the Vienna Center for Experimental Economics, which uses ORSEE for subject pool management (Greiner 2015). Experiments were programmed in Z-Tree (Fischbacher 2007). In total, the study consisted of 7 sessions with 20 groups and a total number of 180 participants. Randomization was performed on the group level. The experiment lasted for about 120 min. On average, participants earned 31.8€ with a minimum of 15.2€ and a maximum of 62.9€ (average earnings by treatment arms: baseline: 26.9€; info sharing: 31.3€; competition: 35.9€; info sharing and competition: 33.0€).

Before the experiment, participants received detailed instructions in the local language (translated instructions in supplement S4) and were given detailed test questions (see supplement S6). Neutral terms were used throughout the experiment. On average, 10.5 of 12 questions were answered correctly. The final payoffs were converted into Euro at an exchange rate of 0.1 (1 token = 0.1€). Participants had to complete a short questionnaire at the end of the experiment after having been informed about their final payoffs.

### Predictions

We derive four testable hypotheses (H). First, both direct transfer costs (within treatment) and competition among trustors (between treatment) are expected to reduce the level of information sharing in the information sharing treatment arms (H1). Second, trustors are expected to behave reciprocally. They are thus more likely to share information about transaction partners who have behaved exceptionally well or poorly and share information with other trustors who have likewise shared information with them in the past (H2). Third, compared to the baseline without information sharing and competition, we expect the costless sharing of information among trustors to lead to higher levels of information exchange, boosting trustworthiness, willingness to trust, and overall payoffs in the market (H3). Fourth, with direct and indirect costs included, trustees are expected to anticipate the increased barriers to information exchange, discouraging them from investing in a good reputation, which, in turn, should lead to reduced levels of trustworthiness, trust, and payoffs (H4). These hypotheses imply the following predictions, which are tested in the same order in the results section:

- *P1a* Within the information sharing treatments (*inf and inf*-*com*), information sharing (I) is more common in rounds with lower direct costs.
- *P1b* Between the information sharing treatments (*inf and inf*-*com*), information sharing (I) is more common in the treatment without competition.
- *P2a* Information sharing (I) increases if a trustee has behaved very well or poorly, i.e. if she has returned more than 150% (Q/P > 1.5~equal share) or less than 100% (Q/P < 1) of the initially sent amount P.
- *P2b* Trustors are more likely to share information (I) the more often other trustors have shared information with them in the past.
- *P3* Sent amounts P, returns on investments Q/P, and payoffs are higher in treatments with information sharing (*inf and inf*-*com*) than in treatments without (*base and com*).
- *P4a* Within the information sharing treatments (*inf and inf*-*com*), sent amounts P, returns on investments Q/P, and payoffs are larger in periods with lower direct costs.
- *P4b* Between the information sharing treatments (*inf and inf*-*com*), sent amounts P, returns on investments Q/P, and payoffs are larger in the treatment without competition.

## Results

### Descriptives

On average, trustors sent 7.75 tokens from their endowment of 10 tokens to their interaction partners, which is a higher share than what was observed in previous studies (e.g. Berg et al. 1995). The majority of trustors (63.9%) decided to send their entire endowment of 10. Similarly, the trustees revealed high levels of trustworthiness. On average, they returned 11.8 tokens, which corresponds to a ROI of 130%. As described above, the identifiability of interaction partners might have led to private reputation effects and direct reciprocity, explaining the overall high levels of trustworthiness and trust observed in the game.

However, betrayals, i.e. returning less than P, also occurred in 13.4% of all interactions. Moreover, some variation in the sending behavior is observable: 13.3% of trustors chose the outside option and did not send anything to the trustee. On average, trustors believed that trustees would return 1.4 times the sent amount P, while the mode was 1.5. We can observe a slight endgame effect in trustees’ responses when approaching the 24th round (see Supplement S2, Figure S5). However, this effect is weak and our results are robust to the exclusion of the final rounds from the analysis. Information sharing among trustors was moderate. Trustors shared information in 44.1% of all possible cases. Trustees believed in 51.9% of all possible cases that information was shared about them.

**Fig. 2:**
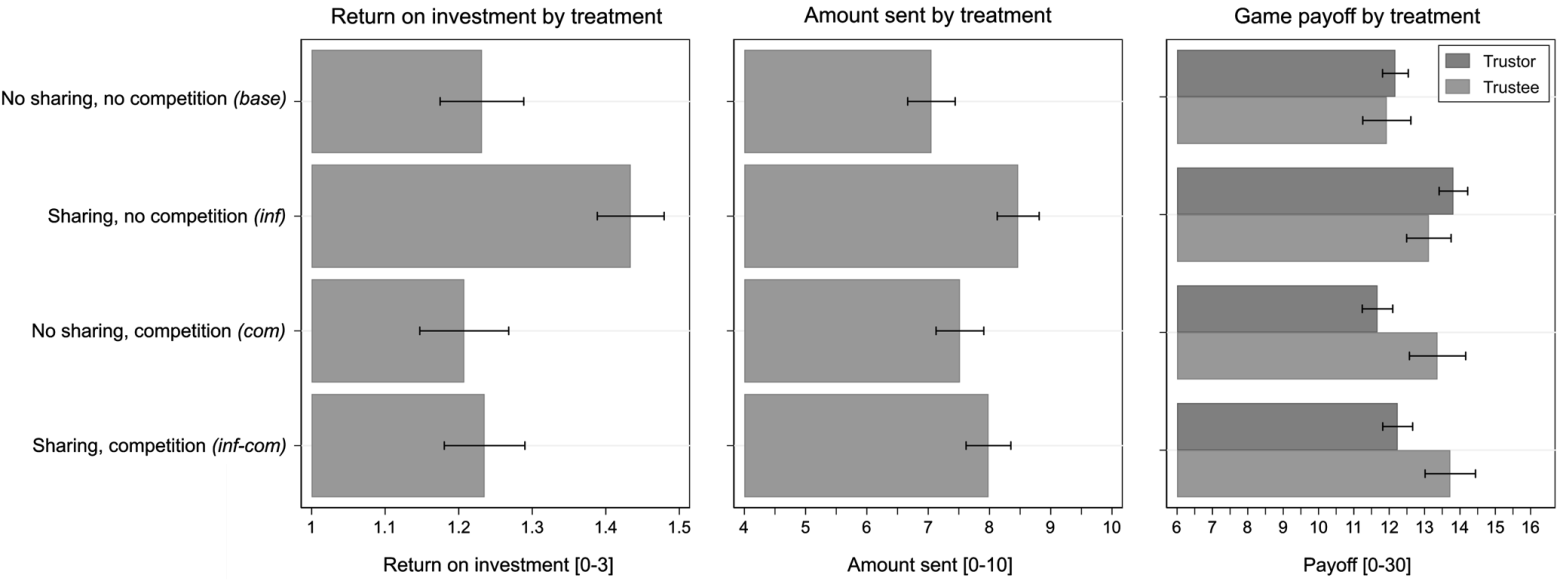
Selected outcomes by treatment

Figure 2 shows the distribution of the four central outcome measures (the ROI, the amount P sent by trustors, and trustors’ and trustees’ payoffs in the game) for each of the four between-subject treatment conditions. Taking no sharing/no competition as the baseline (*base)*, the left panel shows that the ROI increased significantly when trustors could share information with each other (*inf*, *p* < 0.01 two-sided *t* test).^2^ However, adding competition (*inf*-*com*) to the sharing condition reduced the ROI to the baseline level, implying that trustees returned less when trustors competed than when trustors did not compete.

Similarly, in the middle panel, we observe higher amounts sent by the trustors for the two treatments with information sharing (*inf and inf*-*com*). However, information sharing with competition (*inf*-*com*) led trustors to send slightly lower amounts than in the information sharing treatment without competition (*inf*). The differences in the middle panel are smaller than those in the left panel: trustors sent higher amounts in the two competition treatments compared to the baseline even though trustees did not behave in a more trustworthy manner. This willingness to take greater risks and to send more tokens may have been due to a motivation to beat the other trustors and win the tournament. The differences in behavior are also reflected in the game payoffs. While payoffs were highest for trustors in the information sharing treatment without competition (*inf*), they were significantly lower in both competition treatments. Trustees, on the other hand, benefitted from the competition among trustors.

### The impact of transfer costs and competition on information exchange

First, we analyze the impact of direct and indirect costs on information exchange (hypothesis H1). Table 2 shows random effect logit models in which the binary information sharing outcome, measuring whether information was shared by a trustor in a round or not, is regressed on the direct and indirect cost variables. The sample is restricted to the information-sharing treatments (*inf and inf*-*com)*. Models (a) and (b) explore the effect of direct transfer costs, modelled in linear and logarithmic form to capture linear and non-linear cost elasticities, respectively. We also tested for other non-linear specifications, but the logarithmic one resulted in the best overall model fit. Model (c) shows the effect of the indirect cost treatment (*inf*-*com*), i.e. one-sided competition among trustors, on information sharing. Finally, model (d) combines the effects of both cost types. All models presented in this and the following sections are robust to controls for direct reciprocity and variations in the model specifications (see supplement S3).

**Table 2:** RE logit models: Effects of indirect and direct costs on information sharing

	Outcome: trustor information sharing			
	(a)	(b)	(c)	(d)
*Treatments*				
Direct costs [0–1]	− 0.355***			
	[0.096]			
Log direct costs		− 0.056***		− 0.056***
		[0.012]		[0.011]
Competition (*inf*-*com*)			− 0.202**	− 0.202**
			[0.091]	[0.083]
*Controls*				
Period	− 0.024***	− 0.022**	− 0.026***	− 0.020**
	[0.009]	[0.010]	[0.009]	[0.010]
# Prior interactions	− 0.014	− 0.012	− 0.006	− 0.012
	[0.015]	[0.015]	[0.016]	[0.015]
Accumulated payoffs	0.001	0.001	0.001	0.001
	[0.001]	[0.001]	[0.001]	[0.001]
Trustor received bonus	− 0.107	− 0.088	− 0.068	− 0.05
	[0.089]	[0.090]	[0.092]	[0.095]
N	726	726	726	726
AIC	492.3	489.541	528.247	484.621

Random effects (RE) logit models accounting for the hierarchical clustering of the data. Coefficients displayed as marginal probability changes calculated at the mean of all covariates. Clustered standard errors in brackets (unit of clustering: matching groups). Models control for session fixed effects. Sample restricted to treatments with information sharing

*P* values: *p ≤ 0.1, **p ≤ 0.05, ***p ≤ 0.01

Controlling for competition, direct costs had a substantial negative effect on information sharing (prediction P1a). Recall that these costs were randomly varied every round in 0.1 increments. According to model (a) an increase in the direct transfer costs by 1 token led to a 35.5% reduction in the probability of information sharing. If we log-transform the cost variable, we observe a non-linearity in the effect of direct costs on the trustors’ willingness to share information.

**Fig. 3:**
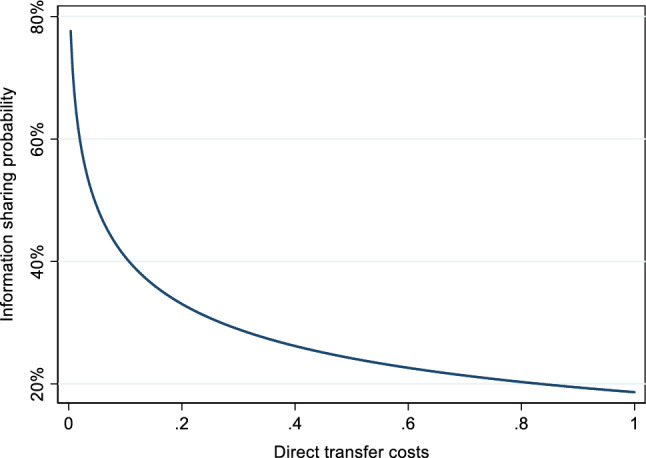
Non-linear effects of direct transfer costs on information sharing. Probability estimates are based on the logarithmic cost function estimated in model (b), Table 2

Figure 3 displays the effect of direct transfer costs on trustors’ estimated probability of sharing information (based on model b). While we observe very high levels of information sharing without any direct costs, there is a sharp decline once a small price for information sharing is introduced. This mirrors the results of Gërxhani et al. (2013) and Abraham et al. (2016). The findings are also in line with Kriss et al. (2016), who show that even small costs diminish the use of communication in coordination games, making coordination failures more likely. In our case, exchange almost completely stopped once the direct transmission costs reached the level of one token (~ 0.1 €). At this point, information was shared in only 8.9% of all interactions.

Table 2 also shows that the one-sided competition treatment (*inf*-*com*) has a strong and significant negative effect on the exchange of reputational information among trustors (prediction P1b). Trustors were 20.2% less likely to share information with others in the competition treatment than in the no competition treatment. This effect is statistically significant and economically meaningful. Once combined in the full model (d), the estimates remain robust. Both direct and indirect costs exerted a substantial effect on the trustors’ willingness to share information with each other.

### Reciprocity and information sharing

Reciprocal motives are expected to influence the level of information sharing in the market (hypothesis H2). Table 3 shows the results of models regressing the binary information sharing outcome on variables that capture the behavior of both trustees and other trustors in the game. In the first two models (a and b), we focus on the effect of trustees’ conduct in the preceding round on information sharing. Model (c) presents the effect of other trustors’ previous information sharing behavior testing whether a trustor is more likely to share information if other trustors have previously shared information.

**Table 3:** RE logit models: the role of reciprocal motives in information sharing decisions

	Outcome: information sharing			
	(a)	(b)	(c)	(d)
Trustee betrayed trustor	0.146***			0.112***
	[0.037]			[0.038]
Trustee returned ROI > 150%		− 0.096***		− 0.064***
		[0.030]		[0.022]
% Info shared by other trustors			0.341***	0.341***
			[0.049]	[0.043]
*Treatments*				
Competition *(inf*-*com)*	− 0.195**	− 0.194***	− 0.065*	− 0.066**
	[0.082]	[0.075]	[0.036]	[0.033]
Log direct costs	− 0.078***	− 0.075***	− 0.044***	− 0.062***
	[0.021]	[0.019]	[0.008]	[0.015]
Observations	628	628	726	628
AIC	420.59	427.349	479.584	410.092

Random effects (RE) logit models accounting for the hierarchical clustering of the data. Coefficients displayed as marginal probability changes calculated at the mean of all covariates. Clustered standard errors in brackets (unit of clustering: matching groups). Models control for session fixed effects. Models additionally control for period effects, accumulated payoffs, whether trustor received a bonus, and number of prior interactions with trustee. Sample restricted to treatments with information sharing. Samples for models (a), (b), and (d) restricted to exchanges where the trustor sent *P* > 0 (n = 628)

*P* values: *p  ≤ 0.1, **p  ≤ 0.05, ***p  ≤ 0.01

We find strong evidence that trustors were willing to sanction misbehavior in the game and to blacklist defectors (negative reciprocity). If a trustee betrayed the trustor, i.e. sent back an amount smaller than the initially sent amount P, the trustors were 14.6% more likely to share information about the trustee with others.^3^ On the other hand, trustors were less likely to share information if they interacted with a generous trustee who returned more than 150% of the initial transfer made. If they interacted with such a “kind” trustee, trustors were 9.6% less likely to share this information. This suggests that the transfer of information about trustees was mostly used as a negative sanctioning device and less as a means to inform others about good types in the trustee population (prediction P2a).

The sharing decision of a trustor is also affected by other trustors’ information sharing behavior in the past (prediction P2b), which we analyze in model (c). The key variable of interest here is the percentage of previous rounds in which other trustors had shared information. As expected, trustors strongly reciprocated the cooperative behavior of their peers. When information was shared by other trustors in all previous rounds, the sharing probability increased by 34.1%, controlling for the costs of information sharing. In additional analyses (see supplement S3.6), we studied trustors’ reactions to the refusal of others to share information with them. Like in the case of trustors’ interactions with trustees, such unkind behavior triggered negative reciprocity, leading to the exclusion of non-cooperative trustors from the information sharing network.

In the final model (d), we estimate the coefficients for all explanatory variables simultaneously. We observe only small changes in the coefficients and all effects remain significant. The results show the importance of reciprocal motives for individual decisions to share information with others and for establishing sustainable reputation mechanisms in a market that suffers from moral hazard problems. The effects of costs on trustors’ willingness to share information remain qualitatively robust even under control for reciprocity. The effect sizes are reduced in the final two models (c and d), as the amount of information sharing by other trustors depended on the treatments. The estimated coefficient for this variable hence captures parts of the cost treatment effects.

### Effects of costs on trustworthiness, trust, and welfare

In this section, we consider the effects of the different treatment conditions on trustworthiness, the willingness to trust, and the payoffs per round for trustees and trustors (hypotheses H3, H4). In Table 4 the main outcome variables are regressed on the treatments. The between-subject treatment variables are included as an interaction to capture the full 2 × 2 experimental design.

**Table 4:** RE models: effects of costs on trustworthiness, trust, and efficiency

	Outcomes: trustworthiness, trust and welfare			
	(a)	(b)	(c)	(d)
	Return on investment by trustee	Amount sent by trustor	Payoff per round trustee	Payoff per round trustor
	[0–3]	[0–10]	[0–30]	[0–30]
*Treatments*				
Sharing (*inf*)	0.260***	2.063***	2.399**	2.206***
	[0.069]	[0.710]	[0.974]	[0.594]
Competition (*com*)	0.059	1.147**	2.330***	0.199
	[0.061]	[0.465]	[0.711]	[0.471]
Sharing and competition *(inf*-*com)*	− 0.275***	− 2.351**	− 2.099	− 2.143***
	[0.091]	[0.938]	[1.386]	[0.690]
Log direct costs	− 0.009*	− 0.045	− 0.065	− 0.133**
	[0.005]	[0.067]	[0.110]	[0.058]
*Controls*				
Period	0.005	− 0.108	− 0.123*	− 0.143***
	[0.006]	[0.081]	[0.070]	[0.046]
# Prior interactions	0.041***	− 0.096	− 0.474**	0.084
	[0.011]	[0.092]	[0.192]	[0.075]
Accumulated payoffs	− 0.003***	0.005	0.027**	0.005*
	[0.001]	[0.006]	[0.012]	[0.003]
Trustor has received bonus in tournament		0.141		− 0.69
		[0.543]		[0.722]
Constant	1.101***	7.232***	12.067***	11.121***
	[0.082]	[0.769]	[1.312]	[0.928]
Observations	1256	1449	1449	1449
R^2^	0.068	0.068	0.052	0.071

Random effects (RE) models accounting for the hierarchical clustering of the data. Linear coefficients with clustered standard errors in brackets (unit of clustering: matching groups). Models control for session fixed effects. Sample for model (a) restricted to exchanges where the trustor sent *P* > 0

*P* values: *p  ≤ 0.1, **p  ≤ 0.05, ***p  ≤ 0.01

When free of costs, the ability to voluntarily share information substantially increased trustworthiness and trust in the market (prediction P3), confirming the findings of many previous studies on the effects of exogenous feedback mechanisms (Keser 2002; Bohnet and Huck 2004; Berg et al. 1995; Bohnet et al. 2005; Bolton et al. 2004; Huck et al. 2012). Trustors’ ability to learn about trustees’ past actions raises incentives for the latter to act more cooperatively. Compared to the baseline reference (*base*), the ROI increased by 26.0 percentage points and the sent amount P by 2.063 tokens. Both trustees and trustors benefitted from the reputation mechanism. Compared to the baseline, their payoffs in the cost-free sharing treatment (*inf*) increased by 2.399 and 2.206 tokens, respectively.

The positive effect of information sharing vanished once trustors were competing with each other (prediction P4b). In the treatment with competition and information sharing (*inf*-*com*), the ROI dropped almost to the level of the baseline treatment without information exchange (model a). The interaction effect of − 0.275 entirely covers the positive effect derived from the ability to share information. Trustors reacted to the changes in returned amounts and sent on average 1.204 tokens fewer than when compared to the treatment with only information sharing (2.351–1.147, significant at α = 0.05).

Compared to the baseline without information exchange (*base*), competition alone had a positive effect of 1.147 on trustors’ sending behavior (*p* < 0.05). Apparently, the prospect of winning the bonus encouraged trustors to take more risks and to send higher amounts, even if their interaction partners did not behave more cooperatively when compared to the baseline without competition. Consequently, while trustors benefitted the most from the ability to share information, trustees benefitted from the rivalry between trustors who sent higher amounts. On average, their payoffs increased by 2.330 tokens in the competition treatment without information sharing (*com*) as compared to the baseline.

To account for the influence of income effects in the models, these estimations were made while controlling for the total accumulated payoff in the game and whether the trustor has received a bonus payment or not. The results are in line with our theoretical expectations, suggesting that the indirect cost of competition reduced information sharing, which subsequently diminished both trustworthiness and trust in the market.

These findings reveal the ambiguous consequences of competition in this setting: As predicted by standard economic theory, increased competition in the market leads to higher exchange. At the same time, however, we observe that competition among trustors leads to less information sharing with negative consequences for trustworthiness and trust in the market. While informational asymmetries are usually assumed to be harmful for competition in markets, our results suggest that there may also be a reverse effect. Competition can harm the reduction of informational asymmetries and the establishment of reputation mechanisms if it is too strong and concentrated among one group of market participants, the trustors in our case.

Like competition, direct transfer costs appear to have a negative effect on trustworthiness, leading to lower returns on investment (prediction P4a). An increase of 1% in direct costs (lin-log model interpretation) decreased the ROI by 0.009 percentage points on average. Similar to competition, direct costs led to a reduction in the sent amount P, but this effect is not statistically significant. Again, it is mainly the trustors who lost from the additional information exchange costs, with their payoffs being significantly lower as compared to the costless information sharing treatment.

### Player perceptions and beliefs

Our argument rests on the assumption that trustees expect information sharing to decrease with increasing information exchange barriers. Such expectations could serve as an explanation for why trustees—who may have feared less punishment—behaved less cooperatively when information sharing was costly. In this section, we test whether changes in the cost environment led to changes in players’ perceptions and beliefs, which may explain the reported behavioral effects.

Table 5 shows the results of models estimating the effect of the treatment conditions on trustees’ beliefs about trustors’ information sharing behavior, trustors’ beliefs about the ROI returned by the trustees, and whether trustors expect betrayal (Q/P < 1). Trustors’ and trustees’ beliefs were measured after they had made their decisions in the investment game. Trustees’ beliefs were operationalized as a binary variable capturing after each round whether they expected that information would be shared about them or not (only in *inf* and *inf*-*com*). Likewise, trustors’ expectations of betrayal were measured as a binary variable. In addition to the control variables, model (a) explicitly controls for whether or not the trustee betrayed (Q/P < 1) the trustor in a round. Thus, we estimate trustees’ beliefs net of their actual behavior in the game. Given the different treatment conditions, did trustees expect more or less information sharing independent of whether they cooperated or defected in this round?

**Table 5:** RE models: treatment effects on players’ beliefs

	Outcome: trustor and trustee beliefs		
	(a)	(b)	(c)
	Trustee beliefs on info sharing [0/1]	Trustor beliefs on ROI [0–3]	Trustor expects betrayal [0/1]
*Treatments*			
Sharing (*inf*)		0.220***	− 0.292***
		[0.041]	[0.059]
Competition (*com*)		0.154***	− 0.180***
		[0.040]	[0.044]
Sharing *and* competition *(inf*-*com)*	− 0.059**	− 0.170***	0.230***
	[0.027]	[0.060]	[0.068]
Log direct costs	− 0.061***	− 0.004	0.013***
	[0.012]	[0.004]	[0.004]
*Controls*			
Period	− 0.001	− 0.003	− 0.007
	[0.005]	[0.006]	[0.008]
# prior interactions	0.002	0.008	0.003
	[0.013]	[0.010]	[0.015]
Accumulated payoffs	− 0.002***	0.001	0.001
	[0.001]	[0.001]	[0.001]
Trustor has received bonus in tournament		− 0.035	0.024
		[0.068]	[0.064]
Trustee has betrayed trustor in this exchange	0.163***		
	[0.061]		
Constant	0.547***	1.162***	0.444***
	[0.134]	[0.047]	[0.082]
Observations	628	1256	1256
R^2^	0.335	0.084	0.09

Random effects (RE) models accounting for the hierarchical clustering of the data. All models were estimated linearly. Clustered standard errors in brackets (unit of clustering: matching groups). Model control for session fixed effects. All model samples restricted to exchanges where the trustor sent P > 0. Sample for model (a), restricted to treatments with information sharing

*P* values: *p  ≤ 0.1, **p  ≤ 0.05, ***p  ≤ 0. 0 1

In line with our expectations, both the competition and direct transfer cost treatments made trustees less likely to expect that information would be shared about them (model a). With competition (*inf*-*com*), trustees were 5.9% less likely to believe that information would be shared about them. An increase in direct costs by 1% further reduced this probability by 6.1%. At the same time, trustees who had betrayed their interaction partner in a round were 16.3% more likely to expect that feedback about them would be shared, mirroring the results on reciprocity reported above.

Likewise, trustors expected higher returns and fewer betrayals when information sharing was possible (*inf*, models b and c). This positive effect of the ability to share information is reduced once trustors compete with each other (*inf*-*com*). At the same time, we observe that competition per se positively affected trustors’ expectations about trustees’ responses. This finding may be due to their tendency to send higher amounts in the first place. Recall that this expectation was not rewarded by trustees, who did not send back higher amounts when trustors competed with each other. The effects of the direct transfer cost variable, although insignificant in model (b), also point in the expected direction. The results suggest that players realized the changes in the information sharing costs and adapted their beliefs and ultimately their behavior accordingly. With higher information exchange barriers, trustees expected less information sharing, which resulted in lower levels of trustworthiness and less trust in the market.

## Discussion and conclusion

The availability of information about the past conduct of transaction partners is an important pre-requisite for the functioning of markets in which the trust problem is paramount. In line with previous research, we show that market participants are willing to share their experiences, which provides incentives to build a good reputation in order to benefit from continued exchanges. The increased trustworthiness of market participants results in a larger willingness to trust and an overall higher market efficiency. Reputation systems, even if they rely on the voluntary sharing of information, can hence contribute to alleviating moral hazard in sequential markets. As we show, information sharing does not occur in isolation but is embedded in social processes and depends to a large extent on group dynamics. Reciprocity is an important behavioral motive explaining agents’ information sharing behavior (Fehr and Gächter 1998; Falk and Fischbacher 2006; Bolton et al. 2004).

We observe a drastic reduction in information sharing once costs are introduced. Even with a small direct transfer cost of 1 token (or 0.1€ which is equivalent to 0.32% of the average earnings), information sharing dropped to 8.9% of all cases. The exchange of information is hence very elastic to increases in direct costs, which mirrors the findings in the previous literature (Gërxhani et al. 2013; Abraham et al. 2016; Kriss et al. 2016).

While direct costs are transfer costs that arise in the information sharing process, indirect costs refer to the loss of potentially valuable private information to a competitor. The impact of our competition treatment on the market is ambivalent: While we observe that competition exerted a positive effect on trustors’ willingness to invest in the market, it simultaneously reduced information sharing by a significant 20.2%. This has negative consequences for the reputation system, which becomes apparent in the reduced level of trustworthiness in the market. Thus, barriers to information exchange are not only important for trustors’ but also for trustees’ behavior, who become less trustworthy as a consequence of increased direct and indirect costs to information sharing.

Complementing previous studies on the effects of competition on trust in markets (Bolton et al. 2008; Huck et al. 2012), we find that there is a strong link between trust and competition. As we show, competition can also exert negative effects on the observed market by creating disincentives for agents to share information and to contribute to reputation systems (see also Huck and Tyran 2007). This effect is particularly strong when competition is concentrated in one group of market participants (the trustors, in our case). Excessive competition or rivalry can lead to the persistence of informational asymmetries and market dysfunctionalities, such as moral hazard problems. Thus, while it is commonly understood that informational asymmetries are harmful for competition, our results show that competition may in turn undermine feedback mechanisms and thus create informational deficits in markets.

An increase in costs, independent of whether they arise directly or indirectly in the sharing process, can result in an information sharing dilemma (Cabrera and Cabrera 2002; Wang and Noe 2010). Although socially beneficial, information may not be shared with others, thus generating inefficiencies. Insufficient information sharing can lead to serious distortions in markets characterized by weak legal environments and low levels of contract enforceability, such as the microfinance example mentioned in the introduction. By imposing binding rules on market participants to disclose their information or by setting strong incentives to do so, institutionalized mechanisms—such as credit bureaus, screening platforms, and externally managed reputation systems—can help to overcome information sharing dilemmas.

We made several design choices that matter for the interpretation of our results. First, while others studied the effects of endogenous competition in markets (Huck and Tyran 2007; Bolton et al. 2008; Huck et al. 2012), we induced competition through an exogenous tournament mechanism. We chose this design as it allowed us to effectively study the influence of competition in a stylized way without unnecessarily adding complexity to our experiment. This design choice comes with the limitation that we eliminate many features of real-world competition in markets, such as prices and the free choice of interaction partners.

Another abstraction of our design is that we allowed only for the transfer of truthful information, which was communicated in a highly standardized and very detailed way. When information was shared, market participants were informed about the entire previous interaction process, which may not always be the case in reality where pieces of information may get lost in the exchange or where only average experiences of past interactions are communicated. We also excluded the possibility of (strategic) lying (Semenova 2006; Crawford 2003; Abraham et al. 2016; Ziv 2006) as well as communication and information exchange between the trustees in the market (Bohnet et al. 2005). While these abstractions come at a cost, they allowed us to focus our experiments on those aspects of the market interactions of primary interest to our study: The impact of voluntary information sharing among trustors and the role of information exchange barriers in influencing trust and reputation building in the market.

Our setting resembles most closely a situation where information exchange is not institutionalized, but occurs mainly in an informal way, such as word of mouth communication. Although we allowed for directed bilateral information sharing in our design, only a few trustors chose to exclude one of their peers from the information exchange. This might have occurred because of the particular set up of our experiment, which did not enable trustors to completely expel competitors from the market or to form collusive agreements, e.g. through price deals (Clarke 2006; Gal-Or 1985). In real markets, there might be incentives for trustors to form coalitions to exclude others from the information sharing network, adding another layer of strategic complexity to the game. The possibility of directed information exchange can hence also result in information cartels with potentially negative implications (Fonseca and Normann 2012; Jacquemin and Slade 1989).

Our findings have several policy implications. First, it is important to acknowledge the role of vertical and horizontal relationships among market participants. It is insufficient to only consider the relationships between trustors and trustees—or lenders and borrowers. The relationships among trustors and among trustees also need to be taken into account as these may affect the market outcome. In particular, competition between the players can create unforeseen dynamics in the market. As we have shown, the costs of information transfer can be essential for trust and the emergence of reputation in markets and they can create substantial welfare losses. These costs are influenced by a variety of factors such as the availability of communication channels, the frequency of contact, the density of an actor’s network, and competition within this network. Reputation building requires an environment that fosters information sharing. To build such an environment in markets prone to moral hazard, structures and mechanisms must be established that reduce information sharing barriers and informational asymmetries.

## Electronic supplementary material

Below is the link to the electronic supplementary material.

Supplementary material 1 (PDF 645 kb)

## Footnotes

1 In the first two sessions costs were fixed at either 0 or 1 to over-sample extreme costs. After this, the experimental protocol was changed to a continuous cost function. We performed robustness checks by excluding both sessions, which did not indicate any problems with these design variations. Session dummies are included to control for any session-specific fixed effects.

2 Standard errors displayed in Fig. 2 are based on t-tests assuming a normal distribution of the tested variables. Wilcoxon-Mann–Whitney rank sum tests on matching group level (n = 20) are reported together with further descriptive statistics in the supplementary material (S2). The exact tests are in line with the findings reported here.

3 In additional analyses not reported in the paper (see supplement S3.6), we test for interaction effects between trustees’ behavior and the different cost treatment conditions. The effect of betrayal is robust independent of barriers to information exchange: If they were betrayed by a trustee in the previous round, trustors were willing to share this information even if this meant bearing direct transfer costs or accepting a loss of valuable information to competitors. This suggests that motives of negative reciprocity or the wish to sanction defectors are very strong in this setting, outweighing in the individual evaluations the potential harm because of direct costs or a loss of competitive advantages in the tournament.
