# Supplementary material for: Information exchange in laboratory markets: competition, transfer costs, and the emergence of reputation
Source: Exp Econ. 2020 Apr 10;24(1):118–42. doi: 10.1007/s10683-020-09652-0 (PMC7954753; doi:10.1007/s10683-020-09652-0)
Supplement: Supplementary file 1 — Supplementary material 1 (PDF 645 kb) [file 10683_2020_9652_MOESM1_ESM.pdf]

Supplementary Online Material

Information Exchange in Laboratory Markets: Competition, Transfer Costs, and the Emergence of Reputation

S1. Literature Overview

S1.1 Reputation Building and Information Sharing

Functioning markets require some level of trust and trustworthiness if contracts are incomplete (Akerlof 1970). The risk of moral hazard and the ensuing low levels of trust, for example in sequential exchanges, can lead to substantial welfare losses. As argued by Schelling (1960) and Kreps and Wilson (1982), economic agents may cooperate – even if opportunities to defect exist – if others can observe or are informed about their behavior. In such situations, players may have an incentive to build a reputation for trustworthiness if future transactions are valuable and sufficiently likely to occur. This may result in cooperation among interaction partners with information about previous interactions serving as a credible signal.

Information about other actors' past behavior can be acquired in different ways: It can be collected in repeated interactions allowing to *privately learn* about the type of the transaction partner. In other situations, agents can rely on publicly available information allowing them to learn from others (Bolton et al. 2004; Huck et al. 2012). The possibility to build a public reputation may serve as a particularly strong disciplining device for market participants who may fear sanctions by others and the exclusion from the market.

Compared to privately learned information, public reputation mechanisms offer greater economic potential because they allow cooperation to be sustained in larger groups. Relatively large-scale exchange systems based on reputation systems existed before formal state specification and enforcement of contracts were in place in many parts of the world. Notable historical examples include trade between northern Africa and Europe in the Mediterranean area (Greif 2006), exchange networks in Paris before the French revolution (Hoffman et al. 1999), in the Italian Alps (Casari 2007), and in Mexican California (Clay 1997).

Market participants can benefit from a good reputation as it allows them, for instance, to charge higher prices (Deephouse 2000; Fombrun & Shanley 1990; Rindova et al. 2005), to attract more productive employees, investors, and customers (Turban & Greening 1997; Fombrun 1996), and to raise competitive barriers (Milgrom & Roberts 1982; Fombrun 1996; Abimbola & Vallaster 2007). The social benefits of reputation have also been shown in several laboratory experiments confirming that reputation mechanisms positively influence trust and efficiency in markets prone to moral hazard (Huck et al. 2012; Keser 2002; Bolton et al. 2004; Bohnet & Huck 2004; Bohnet et al. 2005).

S1.2 Reciprocity

Information sharing is an interaction, which is strongly influenced by reciprocity (Fehr & Gächter 1998; Falk & Fischbacher 2006). As shown in previous research, humans are willing to reward kind actions and to punish unkind ones, even if this is costly for them (Güth et al. 1982; Güth 1995; Camerer & Thaler 1995; Chaudhuri 2011). Accordingly, individuals may sanction defectors by informing others about their misbehavior. In the literature on information sharing in credit markets, this form of negative reciprocity is referred to as black listing (Pagano & Jappelli 1993; Brown & Zehnder 2007). White listing, on the other hand, is the sharing of information about good transaction partners with the intention to reward their good conduct (positive reciprocity). Both forms allow other market participants to choose their transaction partners more carefully. Naturally, also the sharing of information by others can evoke reciprocation.

Bolton et al. (2014) document reciprocity in feedback giving on eBay (see also Bolton et al. 2011 and Diekmann et al. 2014). Abraham et al. (2016) find that trustors are more likely to transfer information about trustees who returned only small amounts (negative reciprocity, black listing). At the same time, trustors who received information from other trustors in the preceding period are significantly more likely to reward this cooperation by sharing their information in the next period. On the other hand, Gërxhani et al. (2013) find no evidence for an effect of the identifiability of the source of information (and hence the possibility to benefit from reciprocal information sharing in the following rounds) on the trustors' willingness to share information. They argue that this may be due to indirect reciprocity, that is, the general willingness to share information with others, as long as some others have shared their information in the past (Anderhub et al. 2002).

S2. Additional Descriptive Statistics

This section provides additional information about the key treatment and outcome variables in our analysis. Figure S1 shows the distribution of the direct transfer cost treatment. The variable takes values from 0-1 and was randomly varied each round except for the first two rounds in which the direct costs were fixed at 0 and 1 explaining the peaks at the two extremes of the cost distribution.

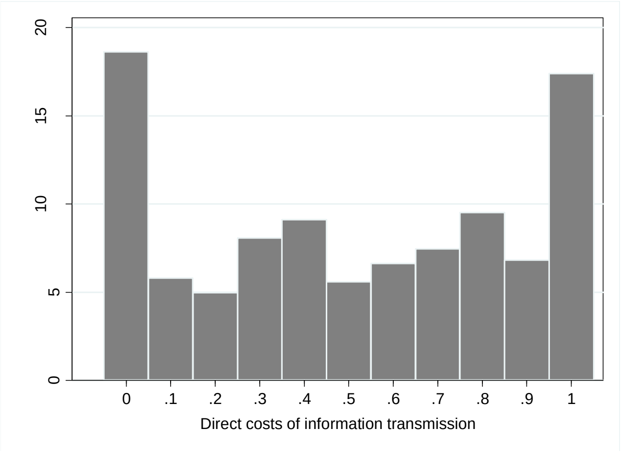

Figure S1 - Distribution of within-subject treatment variable:
Direct costs of information transmission

Figure S2 shows the overall distribution of two of our central outcome measures, the amount P sent by trustors and the ROI (dashed line indicates betraying threshold). Clearly, the focal point was to return 150% of the sent amount. This results in an equal split of total earnings if the trustor sent 10 token to the trustee. Nevertheless, some variation is visible, allowing us to test for the role of the different treatment conditions in changing both trustees' and trustors' behavior. Interestingly, we find that some trustees sent back 3 times the sent amount. One possible explanation for these extraordinarily high returns may be that trustees wanted to make up for prior defections and to send strong signals of trustworthiness.

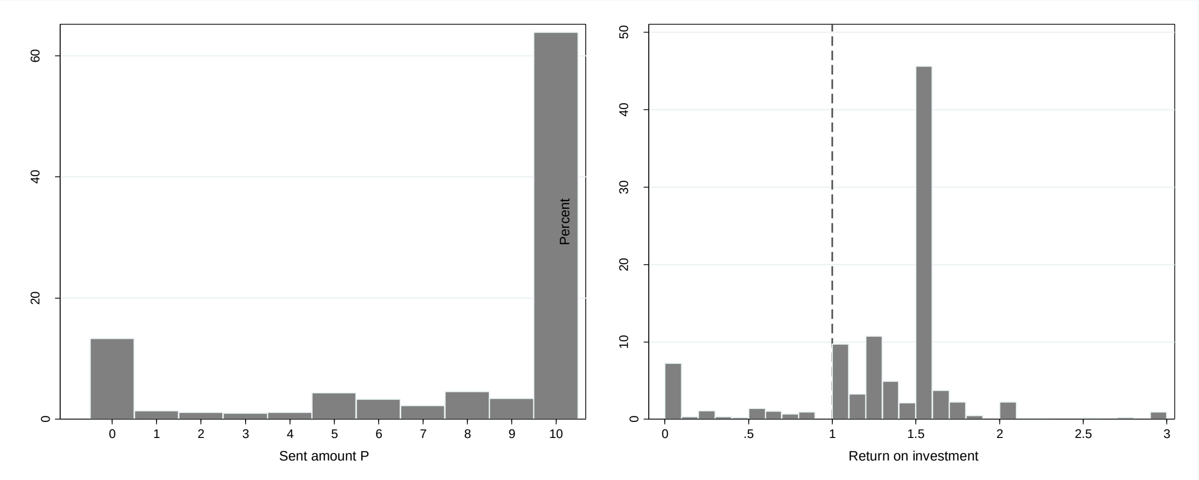

Figure S2 – Distribution of sent amount P and trustees' return on investment (Q/P)

Figure S3 present the distribution of the sent amount P by the different between subject treatments. Overall, we observe less variation and higher sent amounts for the two information sharing treatments (with and without competition). In all four treatments some trustors chose the outside option of sending zero tokens.

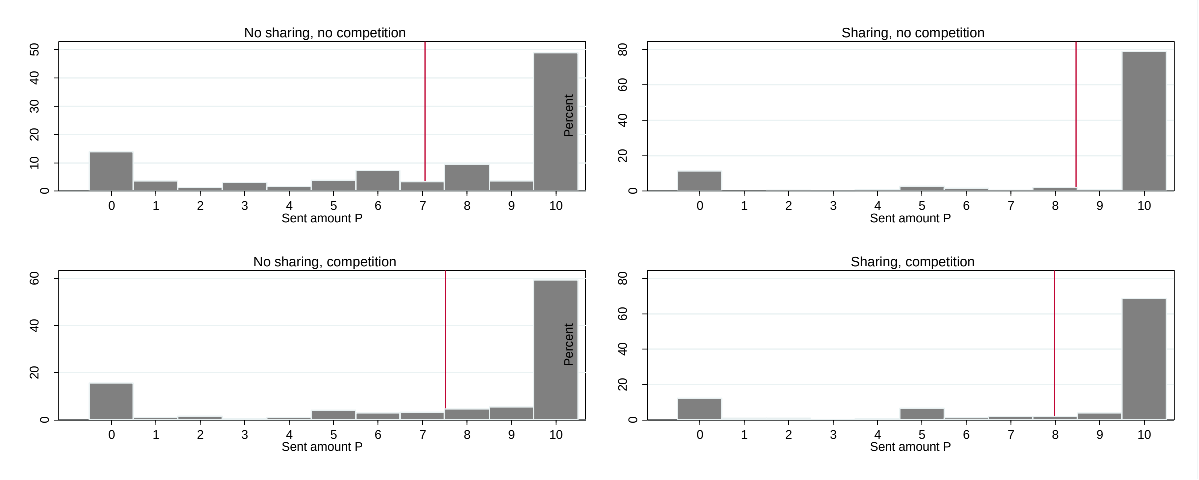

Figure S3 – Distribution of sent amount P by treatment condition

Similarly, Figure S4 shows the distribution of the ROI (Q/P) for the four treatment arms. Here, in particular, the sharing, no competition treatment stands out with overall substantially higher resent amounts and less variation in the resending behavior.

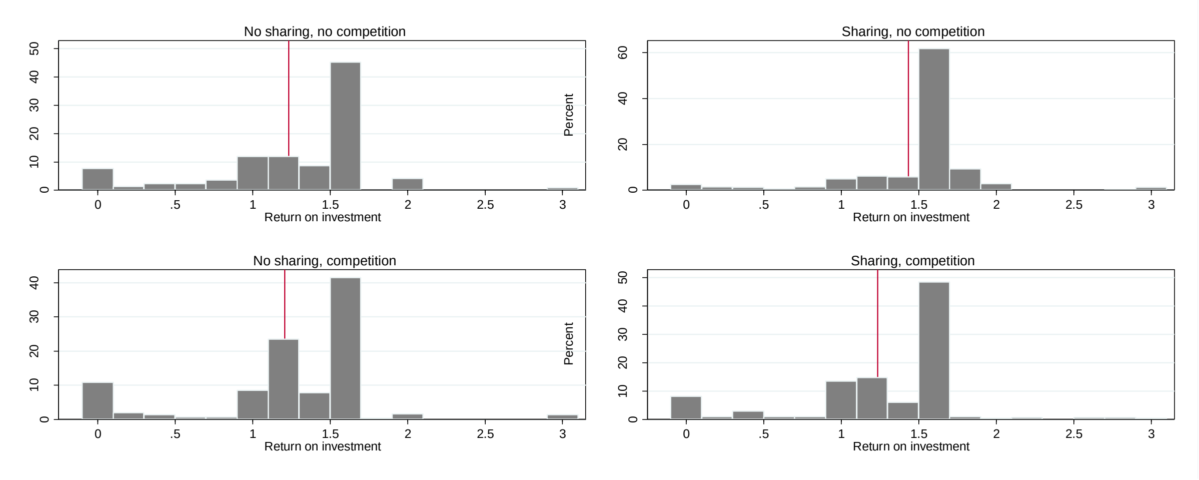

Figure S4 - Distribution of ROI (Q/P) by treatment condition

Figure S5 shows the distribution of the ROI over time for the different treatment conditions. Also in this illustration, it is clearly visible that trustees in the sharing, no competition treatment returned on average the highest percent of the sent amount P. When the game approaches the 24th round, we observe a slight endgame effect in the resending behavior in the sharing, competition treatment.

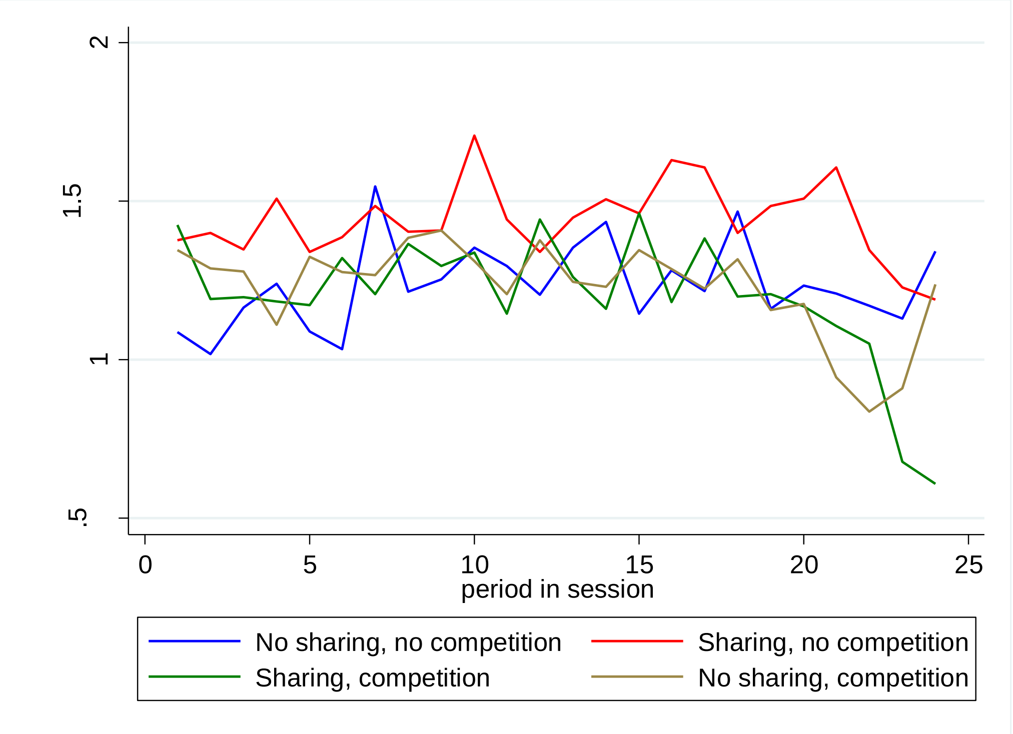

Figure S5 – Distribution of ROI (Q/P) over time by treatment condition

Table S1 provides further summary statistics for the outcomes considered in our analysis. Again, we present the statistics separately for the different between treatment arms. Whereas we observe various differences between the different treatment conditions, the second treatment "sharing, no competition" clearly stands out again. Players in this treatment are more willing to share information as compared to "sharing with competition". Furthermore, trustors send on average

higher amounts and trustees are less likely to betray and return overall higher amounts. The greater levels of trust result in an overall higher payoff for the players. Interestingly, while we find substantial differences in the height of the sent amount P, we do not observe any major differences in trustors' probability to send a positive amount. This suggests that in all treatments, even if trust levels are low, trustors are willing to invest a small share of their endowment into the matched trustee potentially to test them or to leave them a window to re-enter the game (which may correspond with the extraordinarily high ROI of 300% reported above).

Table S1 – Summary statistics of outcome variables by between-subject treatment status

		**Between-subject treatments**							
		**No info sharing No competition**		**Info sharing No competition**		**No info sharing Competition**		**Info sharing Competition**	
	Range	Mean	SD	Mean	SD	Mean	SD	Mean	SD
Information sharing	0/1	-	-	0.191	(0.39)	0.103	(0.30)	-	-
Amount sent P	0-10	7.053	(3.74)	8.468	(3.31)	7.518	(3.78)	7.984	(3.54)
Trustor sent positive amount	0/1	0.287	(0.45)	0.296	(0.46)	0.281	(0.45)	0.292	(0.45)
Trustee return on investment	0-3	1.232	(0.51)	1.434	(0.41)	1.208	(0.54)	1.235	(0.50)
Betraying (Q<P)	0/1	0.493	(0.50)	0.408	(0.49)	0.469	(0.50)	0.456	(0.50)
Game payoffs	0-30	8.035	(7.12)	8.979	(7.61)	8.345	(7.81)	8.656	(7.70)
Trustor's beliefs	0-3	1.269	(0.38)	1.446	(0.24)	1.427	(0.41)	1.365	(0.28)
Minimum acceptable amount	0-3	1.167	(0.29)	1.294	(0.25)	1.109	(0.41)	1.138	(0.32)
Trustee`s beliefs	0/1	-	-	0.556	(0.50)	0.480	(0.50)	-	-

Table S2 shows two-sample Wilcoxon rank-sum (Mann-Whitney) tests, analyzing differences in the trustworthiness and trust between the different treatment arms. Unlike the test statistics reported in section 4.1, these tests are performed on matching group level. In line with our other findings, they show higher returns on investment and higher sent amounts with information sharing in the no competition treatment arms (1). At the same time, competition significantly reduces the positive effect of voluntary information exchange on trustees' resending behavior (2) and makes trustors more likely to send higher amounts, if there is no information sharing (Prob > |z|with 0.1172 insignificant) (3).

Table S2 – Two-sample Wilcoxon rank-sum (Mann-Whitney) tests

				Return on investment by trustee Q/P	Amount sent by trustor P
(1)	No competition	No sharing	Rank sum	17 (5)	19 (5)
		Sharing	Rank sum	38 (5)	36 (5)
		Rank-Sum Test	z test value	-2.193	-1.776
			Prob > |z|	0.0283**	0.0758*
(2)	Competition	No sharing	Rank sum.	31 (5)	29 (5)
		Sharing	Rank sum.	24 (5)	26 (5)
		Rank-Sum Test	z test value	0.731	0.313
			Prob > |z|	0.4647	0.7540
(3)	Sharing	No competition	Rank sum	38 (5)	32 (5)
		Competition	Rank sum	17 (5)	23 (5)
		Rank-Sum Test	z test value	2.193	0.940
			Prob > |z|	0.0283**	0.3472
(4)	No Sharing	No competition	Rank sum	25 (5)	20 (5)
		Competition	Rank sum	30 (5)	35 (5)
		Rank-Sum Test	z test value	-0.522	-1.567
			Prob > |z|	0.6015	0.1172

Numbers in parenthes show the number of groups per treatment arm. Expected rank under H0 is 27.5 in all tests. P-values: * p≤0.1, ** p≤0.05, *** p≤0.01

S3. Model Variations and Extensions

In this section, we vary the main model specifications to explore further the underlying mechanisms influencing players' decision-making and to test for the robustness of our findings. In particular, extending our main models, we more directly control for direct reciprocity in influencing the outcome of the game (S3.1), estimate models without any additional controls (S3.2), separate our main models for the competition and non-competition treatments (S3.3), provide further evidence on the competition treatment (S3.4), present models with alternative outcome indicators (S3.5). and estimate additional models, which further explore the role of reciprocity in information sharing (S3.6).

S3.1 Controlling for Direct Reciprocity

Table S3 and Table S4 extend the main models in the paper (Table 2 and 4) by additionally including a dummy indicating whether the trustor was betrayed by the trustee in the past interaction prior to the current round. Since the betrayal variable is itself an outcome of our treatments, it was not included it in our main models. Trustor's information sharing is not significantly influenced by the past history of interactions with the trustee, but only by trustee's behavior in the current round.

Table S3 – RE logit models: Effects of costs on information sharing controlling for direct reciprocity

	Outcome: Trustor information sharing			
	(a)	(b)	(c)	(d)
**Treatments**				
Direct costs [0-1]	-0.357***			
	[0.098]			
Log direct costs		-0.056***		-0.056***
		[0.012]		[0.010]
Competition (*inf-com*)			-0.202**	-0.201**
			[0.092]	[0.083]
**Direct reciprocity**				
Period	-0.024***	-0.021**	-0.026***	-0.020**
	[0.009]	[0.010]	[0.009]	[0.010]
# prior interactions	-0.013	-0.012	-0.007	-0.012
	[0.016]	[0.015]	[0.016]	[0.015]
Trustor was betrayed by trustee in previous interaction	-0.017	-0.015	0.005	-0.013
	[0.042]	[0.046]	[0.042]	[0.046]
N	726	726	726	726
AIC	492.111	489.416	528.232	484.524

Note: Random effects (RE) logit models accounting for the hierarchical clustering of the data. Coefficients displayed as marginal probability changes calculated at the mean of all covariates. Clustered standard errors in brackets (unit of clustering: matching groups). Models control for session fixed effects, accumulated payoffs and whether the trustor received a bonus payment. Sample restricted to treatments with information sharing. P-values: * p≤0.1, ** p≤0.05, *** p≤0.01

Trustworthiness and the willingness to trust (Table S3) significantly depend on trustee's behavior in the past interaction. A trustee who betrayed a trustor in the past interaction sends back on average smaller returns on investment (if given a chance), which points towards a persistence of defective behavior in the game. At the same time, trustors send on average 4.755 tokens less to

trustees who have betrayed them in the past round, resulting in lower average payoffs for both player types, trustees and trustors. Importantly, even under control for this measure of direct reciprocity, the treatment effects are robust. While direct reciprocity is important in our game, it has not affected (i) the positive impact of the possibility to share information and to build a public reputation, and (ii) the negative impact of direct and indirect costs on information sharing and the outcomes of the market.

Table S4 – RE models: Effects of costs on trustworthiness, trust, and payoffs controlling for direct reciprocity

	**Outcomes: Trustworthiness, trust and welfare**			
	(a) Return on investment by trustee [0-3]	(b) Amount sent by trustor [0-10]	(c) Payoff per round trustee [0-30]	(d) Payoff per round trustor [0-30]
**Treatments**				
Sharing (*inf*)	0.252***	1.787***	1.574*	1.974***
	[0.039]	[0.497]	[0.888]	[0.435]
Competition (*com*)	0.033	1.081***	2.102***	0.182
	[0.034]	[0.417]	[0.806]	[0.353]
Sharing & competition *(inf-com)*	-0.234***	-1.981***	-1.473	-1.963***
	[0.053]	[0.651]	[1.230]	[0.478]
Log direct costs]	-0.013**	-0.059	-0.026	-0.128***
	[0.005]	[0.056]	[0.095]	[0.043]
**Direct reciprocity**				
Period	0.003	-0.017	-0.088	-0.081*
	[0.006]	[0.055]	[0.059]	[0.042]
# prior interactions	0.052***	0.083	-0.179	0.194***
	[0.011]	[0.085]	[0.186]	[0.065]
Trustor was betrayed by trustee in previous interaction	-0.397*** [0.063]		-6.712*** [0.752]	
Constant		-4.756***		-2.869***
		[0.443]		[0.235]
Observations	1.147***	7.942***	13.957***	11.647***
R^2	[0.068]	[0.749]	[1.510]	[0.709]

Note: Random effects (RE) models accounting for the hierarchical clustering of the data. Linear coefficients with clustered standard errors in brackets (unit of clustering: matching groups). Models control for session fixed effects, accumulated payoffs and whether the trustor received a bonus payment. P-values: * p≤0.1, ** p≤0.05, *** p≤0.01

S3.2 Models without Additional Controls

Table S5 and S6 re-estimate the main models excluding any control variables from the specification. The aim here is to estimate models which do not control for any game dynamics, which might potentially confound the effect estimates. Even if estimated without additional controls, all models remain perfectly robust.

Table S5 – RE logit models: Effects of costs on information sharing without controls

	**Outcome: Trustor information sharing**			
	(a)	(b)	(c)	(d)
**Treatments**				
Direct costs [0-1]	-0.364***			
	[0.102]			
Log direct costs		-0.060***		-0.060***
		[0.012]		[0.009]
Competition (*inf-com*)			-0.202**	-0.198***
			[0.079]	[0.068]
N	726	726	726	726
AIC	517.322	506.019	554.853	504.355

Note: Random effects (RE) logit models accounting for the hierarchical clustering of the data. Coefficients displayed as marginal probability changes calculated at the mean of all covariates. Clustered standard errors in brackets (unit of clustering: matching groups). Models control for session fixed effects. Sample restricted to treatments with information sharing. P-values: * p≤0.1, ** p≤0.05, *** p≤0.01

Table S6 – RE models: Effects of costs on trustworthiness, trust, and payoffs without controls

	**Outcomes: Trustworthiness, trust and welfare**			
	(a) Return on investment by trustee [0-3]	(b) Amount sent by trustor [0-10]	(c) Payoff per round trustee [0-30]	(d) Payoff per round trustor [0-30]
**Treatments**				
Sharing (*inf*)	0.249***	2.322***	2.582**	2.175***
	[0.060]	[0.720]	[1.169]	[0.588]
Competition (*com*)	0.027	1.506***	2.768***	0.216
	[0.056]	[0.406]	[0.763]	[0.475]
Sharing & competition (*inf-com)*	-0.244***	-2.463***	-2.530*	-2.363***
	[0.080]	[0.915]	[1.536]	[0.724]
Log direct costs	-0.012***	-0.07	-0.046	-0.114***
	[0.004]	[0.068]	[0.120]	[0.036]
Constant	1.031***	6.137***	11.753***	10.350***
	[0.069]	[0.660]	[1.396]	[0.711]
Observations	1256	1449	1449	1449
R^2	0.046	0.052	0.034	0.056

Note: Random effects (RE) models accounting for the hierarchical clustering of the data. Linear coefficients with clustered standard errors in brackets (unit of clustering: matching groups). Models control for session fixed effects. P-values: * p≤0.1, ** p≤0.05, *** p≤0.01

S3.3 Separate Models for Competition vs. Non-Competition Treatments

Aside of introducing competition, the tournament mechanism also leads to an increase in income for trustors. Although, it is unlikely that the observed treatment effects are merely driven by the artificially induced jump in the incomes, we re-estimate the main models in this section separately for treatments with and without competition to show that the results for endogenous information sharing and the influence of direct costs hold for both arms of the competition treatment. Table S7 shows the results for the main game outcomes (information sharing, ROI, send amount P, trustee payoffs, and trustor payoffs) separated for competition vs. non-competition treatments.

Table S7 – RE models: Effects of treatments on market outcomes for competition vs. non-competition

	**Non-competition treatments (*base & inf*)**				
	(a)	(b)	(c)	(d)	(a)
	Return on investment by trustee [0-3]	Amount sent by trustor [0-10]	Payoff per round trustee [0-30]	Payoff per round trustor [0-30]	Return on investment by trustee [0-3]
**Treatments**					
Sharing (*inf*)		0.309***	1.077**	1.198***	2.360***
		[0.039]	[0.469]	[0.382]	[0.405]
Log direct costs	-0.106***	-0.025***	-0.210***	-0.208***	-0.191***
	[0.031]	[0.007]	[0.053]	[0.076]	[0.032]
N	363	632	723	723	723
R^2	-	0.087	0.177	0.225	0.116
	**Competition treamtnents (*com & inf-com*)**				
	(a)	(b)	(c)	(d)	(a)
**Treatments**					
Sharing (*inf-com*)		-0.142*	-1.090**	-0.608	-1.547***
		[0.086]	[0.435]	[1.038]	[0.447]
Log direct costs	-0.029***	0.008**	0.055	0.076	0.05
	[0.002]	[0.003]	[0.077]	[0.149]	[0.031]
N	363	624	726	726	726
R^2	-	0.077	0.094	0.077	0.086

Note: Random effects (RE) logit models accounting for the hierarchical clustering of the data. Coefficients displayed as marginal probability changes calculated at the mean of all covariates. Clustered standard errors in brackets (unit of clustering: matching groups). Models control for session fixed effects. Additional controls included, but not displayed: period effects, number of interactions between trustor and trustee, accumulated payoffs and whether the trustor received a bonus payment. P-values: * p≤0.1, ** p≤0.05, *** p≤0.01

S3.4 Further Evidence on Competition Treatment

In this sub-section we test how different positions in the ranking affected trustor's behavior. The models in Table S8 analyze how the ranking position of trustors who participated in the tournament (competition treatment) affected their willingness to share information with others, their willingness to trust reflected in the amount sent to trustees, and their game payoffs.

We do not observe any statistically significant effect of the ranking position in the last round on information sharing behavior, i.e. all trustors in the competition treatment shared similarly little information. Trustors with a higher ranking, on the other hand, were more likely to send higher amounts P to trustees ($p<0.05$) and benefited of a higher overall payoff from the game.

Table S8 – RE models: Effects of tournament ranking position on trustors' behavior

	(a) Trustor info sharing [0/1]	(b) Amount sent by trustor [0-10]	(c) Payoff per round trustor [0-30]
**Placement in tournament**			
3rd place in ranking (reference)			
1st place in ranking (round t-1)	-0.039**	2.015***	3.852***
	[0.019]	[0.500]	[0.306]
2nd place in ranking (round t-1)	-0.015	0.645	2.375***
	[0.051]	[0.508]	[0.310]
Observations	363	726	726
R^2	-	0.146	0.226

Note: Random effects (RE) models accounting for the hierarchical clustering of the data. Linear coefficients with clustered standard errors in brackets (unit of clustering: matching groups). Models control for session fixed effects. Additional controls included, but not displayed: direct costs, period effects, number of interactions between trustor and trustee, accumulated payoffs and whether the trustor received a bonus payment. Sample restricted to treatments with competition. P-values: * p≤0.1, ** p≤0.05, *** p≤0.01

S3.5 Models with Alternative Outcome Indicators

Table S9 presents the findings of the treatment effects on trustees' and trustors' behavior in the investment game for two alternative binary trustworthiness and trust indicators: Whether the trustee betrayed the trustor by returning less than the sent amount P and whether the trustor decided to send a positive amount to the trustee in the first place (P>0). All findings remain qualitatively robust with the use of those alternative outcomes.

Compared to a baseline without information sharing, the possibility to exchange information between trustors reduced the probability for betrayal by 18.3% and for trustors to engage in the exchange by 10.4% (not significant). This positive effect is reduced once competition is introduced leading to a significant reduction in trustworthiness and trust in the market. Likewise, an increase in direct information transfer costs leads to an increase in the betrayal risk and a reduced willingness to send a positive amount, but these effects are not statistically significant.

Table S9 – RE logit models: Effects of costs on trustworthiness and trust with alternative outcomes

	**Outcomes: Trustworthiness and trust**	
	Trustee betrayal ROI < 1 [0/1] (a)	Trustor sent positive amount P>0 [0/1] (b)
**Treatments**		
Sharing (*inf*)	-0.188***	0.107
	[0.061]	[0.073]
Competition (*com*)	-0.063	0.04
	[0.043]	[0.030]
Sharing & competition *(inf-com)*	0.163**	-0.116*
	[0.076]	[0.062]
Log direct costs	0.008	-0.003
	[0.006]	[0.006]
Observations	1256	1449
AIC	966.538	1066.847

Note: Random effects (RE) logit models accounting for the hierarchical clustering of the data. Coefficients displayed as marginal probability changes calculated at the mean of all covariates. Clustered standard errors in brackets (unit of clustering: matching groups). Models control for session fixed effects Additional controls included, but not displayed: direct costs, period effects, number of interactions between trustor and trustee, accumulated payoffs and whether the trustor received a bonus payment. P-values: * p≤0.1, ** p≤0.05, *** p≤0.01

S3.6 Further Tests on the Role of Reciprocity in Information Sharing

Table S10 shows logit models in which the binary information sharing outcome is regressed on the different treatment conditions. Mainly, we are interested here whether costs diminish the effects of negative reciprocity on trustors' willingness to share information with each other. For this, we interact the dummy measuring if a trustor was betrayed in the previous round with the competition treatment (model b) and the direct transfer cost treatment (model c). Model a shows the baseline result from Table 3 in the main text.

While, we observe that both direct and indirect costs reduce information sharing in the baseline model (a), betraying has a positive effect. The betraying effect remains significant in the second model. The interaction with competition, on the other hand, is insignificant suggesting that players are also willing to sanction betrayers about others if this is costly for them. Also in the final model (c) the interaction between betraying and direct costs is insignificant. However, in this model the main effect of betraying is also considerably reduced, even though it still points in the expected direction.

In the model displayed in Table S11, the binary information sharing outcome is regressed on a variable which takes the value one if none of the other trustors has shared information with the trustor in any of the previous rounds. Here, we are interested how the peers' refusal to share information, even if ego shared information in the past, affects ego's willingness to share another time information. For this, we condition our sample on those cases for which the trustor has sent some information in the past, but for which this positive sharing behavior was not reciprocated by

the fellow trustors. As this condition significantly reduces the sample size, we focus on simple correlations between the two main variables of interest here.

Table S10 – RE Logit models: The role of reciprocal motives in information sharing decisions

	**Outcome: Information sharing**		
	(a)	(b)	(c)
Betraying	0.146***	0.130*	0.112
	[0.037]	[0.067]	[0.110]
Competition (*inf-com*)	-0.195**	-0.214***	-0.207***
	[0.082]	[0.079]	[0.067]
Betraying*competition		0.025	
		[0.091]	
Log direct costs	-0.078***	-0.077***	-0.079***
	[0.021]	[0.020]	[0.020]
Betraying*log direct costs			0.075
			[0.125]
Period	-0.019**	-0.016***	-0.017**
	[0.008]	[0.005]	[0.007]
Observations	628	628	628
AIC	420.59	420.912	430.079

Note: Random effects (RE) logit models accounting for the hierarchical clustering of the data. Coefficients displayed as marginal probability changes calculated at the mean of all covariates. Clustered standard errors in brackets (unit of clustering: matching groups). All models control for session fixed effects. Sample restricted to treatments with information sharing. P-values: * p≤0.1, ** p≤0.05, *** p≤0.01

Table S11 – RE Logit models: The role of reciprocal motives in information sharing decisions

	**Outcome: Information sharing**
	(a)
No info shared by others before	-0.368**
	[0.158]
Observations	658
AIC	516.869

Note: Random effects (RE) logit models accounting for the hierarchical clustering of the data. Coefficients displayed as marginal probability changes calculated at the mean of all covariates. Clustered standard errors in brackets (unit of clustering: matching groups). Models control for session fixed effects. Sample restricted to treatments with information sharing. P-values: * p≤0.1, ** p≤0.05, *** p≤0.01

The results clearly confirm the findings from the main analysis. If a trustor has shared her experiences with other trustors in the past, but these did not reciprocate this behavior, then a trustor's willingness to share information is reduced by a significant 36.8%. This suggests that negative reciprocity as a behavioral motive does not only play a role for interactions between trustors and trustees, but also for the interactions among the trustors. Information sharing mechanisms are hence only functional if all members of the information sharing network are equally willing to contribute by sharing their previous experiences in the market.

S4. Translated Experimental Instructions (English)

THANK YOU FOR YOUR PARTICIPATION!

Please do not talk to other participants during the experiment!

Dear participants,

Thank you for your participation in our experiment. In the experiment, we are interested in decisions made in groups. For the success of our study it is very important that you read the following instructions carefully.

The experiment will take about 2 hours of your time. In the experiment you can earn money whereas the total amount of your payoff depends on your decisions and the decisions of other participants. After the experiment, you will be paid individually and anonymously in cash. During the experiment we do not speak of Euro, but of token. At the end of the experiment, the token earned will be converted according to the following exchange rate:

10 token = 1.00 Euro

Please take your time when reading the instructions and making your decisions. You <u>cannot</u> influence the duration of the experiment by making quick decisions. Also, remember that the amount of your payout depends on your choices. Before starting the experiment, you will be provided with some test questions to help you check whether you have understood the instructions. For each correctly answered test question you get 1 extra token. At no time during the experiment will the identity of the participants and your payoffs be revealed. Your anonymity towards the researchers and the other participants is guaranteed.

If you have any questions while reading the instructions, please raise your hand. One of the assistants will come to you and answer your question privately. Please do not communicate with other participants and ask your questions quietly. Communication between the participants leads to exclusion from the experiment. After the start of the experiment no further questions may be asked

Study procedure

At the beginning of the experiment, you are randomly assigned to a group of 9 participants (hereafter we speak of players). Each player is given a unique ID and one of two possible roles: Role A or Role B. Each of the groups consists of 3 players with the role A (A-player) and 6 players with the role B (B-player). The group composition, the role distribution and the IDs of the participants do not change throughout the entire experiment: You interact with the same participants and keep your individually assigned role. The experiment lasts at least for 24 rounds. After the 24th round it ends with a probability of 1/2 (= 50%) in each additional round. Each of the minimum 24 identical rounds consists of different parts, which are presented below

Part 1: In each round, each of the 3 A-players is randomly assigned to a B-player, with whom she/he interacts in the round. At the beginning of the round, the A-player is informed about the identity of the assigned B-player (B1, B2, B3, B4, B5 or B6) and the B-player learns the identity of the assigned A-player (A1, A2 or A3). The 3 B-players, who are not assigned to an A-player, pause for one round. Instead of interacting with an A-player, the pausing B-players are shown arithmetic tasks they can solve. To solve the tasks, the B-players have 30 seconds. They receive 3 additional tokens for each solved task. The following parts of the experiment refer only to A and B-players who interact with each other.

Part 2: Each A-player receives an initial endowment of 10 token per round. In the second part of the round, the A-player can send a portion of the initial endowment to the matched B-player. At maximum, the A-player can send the entire endowment of 10 token. On the way to the B-player, the transferred amount (in the following X) is tripled (3X).

3rd part: In the next step, the B player can return any part of the tripled sent amount (3X). The amount returned (In the following Y) cannot be greater than the tripled transferred amount (0≤Y≤3X).

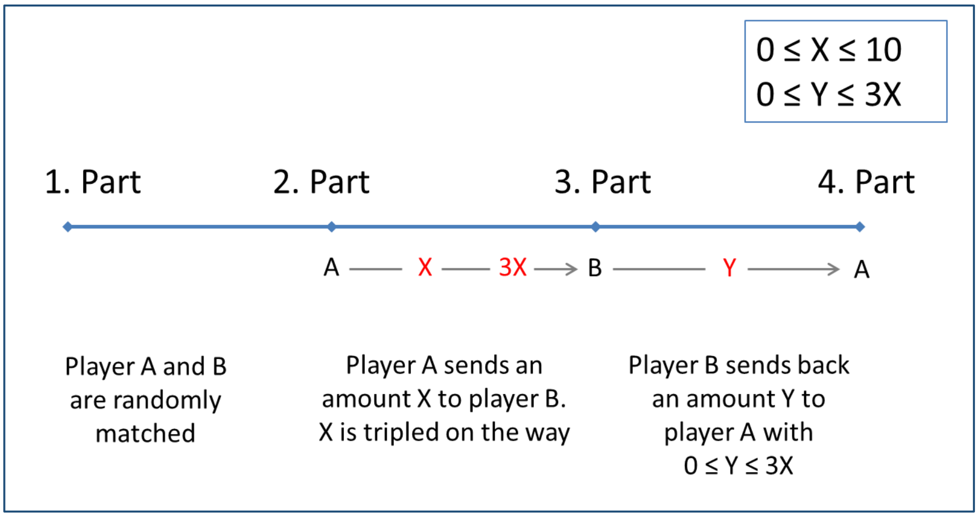

4. Part: The A-player is informed about the amount Y returned by the B-player. Both players are informed about their payout in tokens from the round as well as the sum of their earning from all previous rounds.

The payoffs of the players in a round are calculated as follows:

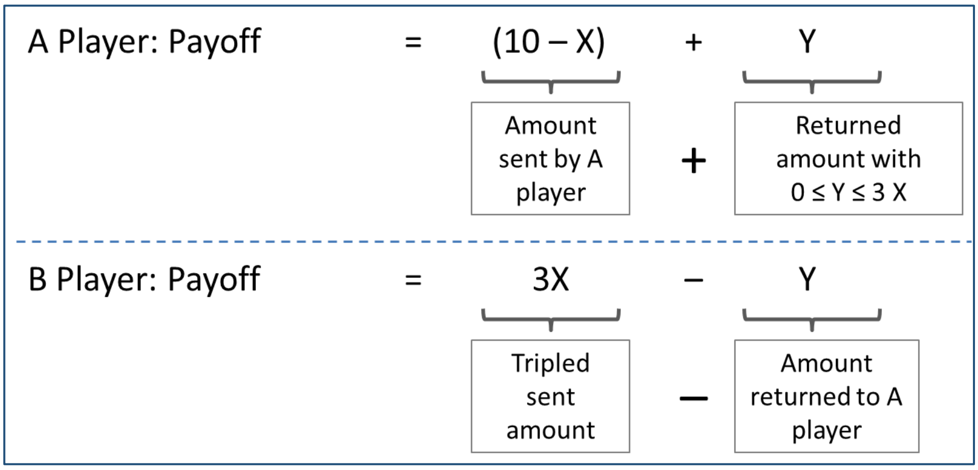

The following instructions are provided only in competition treatments

Ranking of the A-players: Based on the sum of the previous payoffs, a ranking of the 3 A-players is created in the 4th part and displayed to the A-players. The A-player with the highest sum of

payoffs in tokens over all previous rounds receives the first rank. The A-player with the second highest sum is ranked second. The A-player with the lowest sum is ranked third. If two A-players have acquired the same sum over all rounds, they are assigned the same rank. After each eight rounds (after the end of the 8th, the 16th and the 24th round), the A-player who is ranked 1st will receive a bonus of 100 tokens (~ 10 €) in addition to his previous payoffs. The player on the second rank receives a bonus of 50 tokens (~ 5 €). The player on the third rank does not receive any bonus. After the 8th, 16th and 24th round, a new ranking list is created for which only the sum of the payoffs from the 8th and 16th round on are considered (without the bonus payments received). The A-players are shown only the sum of the payoffs of the previous rounds since the last bonus payout. For the B-players, no ranking list and bonus payments exist.

The following instructions are provided only in information sharing treatments

Part 5: At the end of each round, A-players can share their experiences from this round with other A-players. The A-players can decide whether they want to share their experiences with none, one or both other A-players. Sharing information with one of the other two A-players costs the communicating A-player an amount C that lies between zero points and one point ($0 \leq C \leq 1$). The amount changes randomly in each round and is communicated to all A-players and B-players at the beginning of the round. If an A-player passes on information to both other A-players, the doubled amount of token (2C) is deducted from his payoff in this round. When information is shared, the receiving A-player(s) receive the following information in the next round:

1. ID of the B-player the information-sharing A-player has played with in the last round (B1-B6)

2. What amount (X) the A-player has sent and the level of the amount after tripling (3X)

3. What amount (Y) the assigned B-player has sent back.

The information shared by other A-players is displayed to the A-players in form of two history tables (a table for each other A-player) in the first part of the next round (see screenshot next page). Each row of the tables represents the transferred information from one of the previous rounds. If no information was shared, this is displayed in the table with the words "no info". **Note**: Sharing information with another A-Player does not mean that this A-Player also wants to share information with you. An information exchange between B-players is not possible.

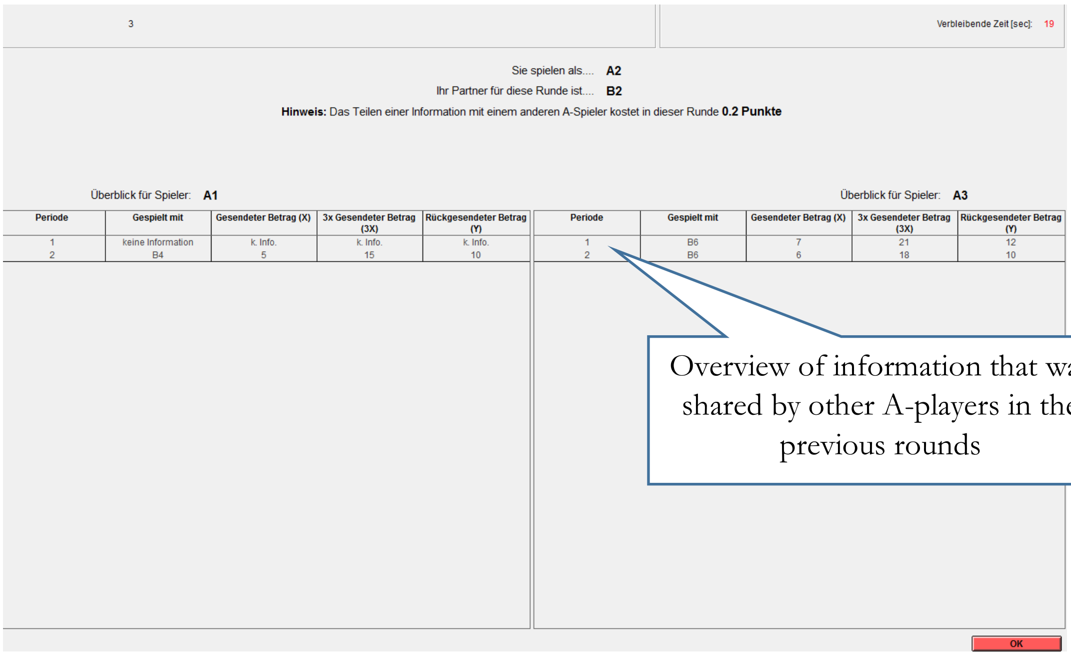

After the 5th part, the subsequent of at least 24 rounds of the experiment starts. During the individual rounds of the experiment, you are repeatedly asked about your expectations regarding the behavior of your interaction partner. If your expectations match the actual behavior of your fellow players, you will receive an additional payout at the end of the experiment.

Take your time to read the instructions again. If you have a question, please raise your hand. On the following page you will find an overview of the instructions. Please keep it in front of you during the experiment.

OVERVIEW OF INSTRUCTIONS

Please keep this overview always in front of you during the entire experiment

In total, 9 player per group, 3 A-players and 6 B-players

At least 24 rounds. Each round consists of 5 parts

After the 24th round the experiment ends with probability 50%

**1. Part**	- A and B players are randomly matched and are informed with whom they interact in the round<br>- A-players receive information shared by others in previous rounds (only in sharing treatment)<br>- Unassigned B-players solve arithmetic tasks and get 3 tokens per correct solution
**2. Part**	- A-players receive 10 tokens per round as initial endowment<br>- A-players can send amount X to assigned B-players (0≤X≤10).<br>- The amount X is tripled on the way to B (3X)
**3. Part**	- B-players can send amount Y back to A-player. Y not greater than 3X (0≤Y≤3X)
**4. Part**	- A and B-players learn the game history and their payoffs as well as the sum of the payoffs from all previous rounds<br>- A-players are informed of their ranking and the ranking of the other A-players<br>- The rank depends on the sum of the payoffs from the previous rounds<br>- After the 8th, 16th, and 24th round, the ranking will start again. The first ranked A-player receives a bonus of 100 token, the second ranked a bonus of 50 token (only in competition treatment)
**5. Part**	- A-players can share their experiences from this round with other A-players (only in sharing treatment)<br>- Sharing information costs an amount C, which varies between 0 and 1 token in each round<br>- The selected A-players are informed with who the information sending A-player interacted with in the last round, which amount (X) was sent, and how much was returned (Y)

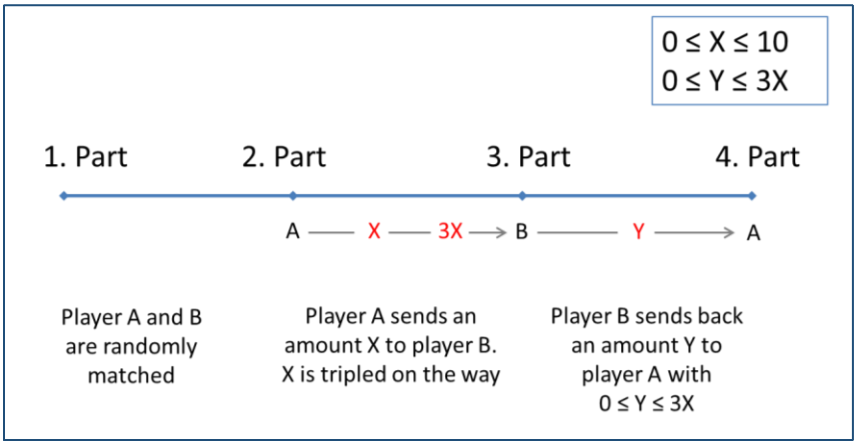

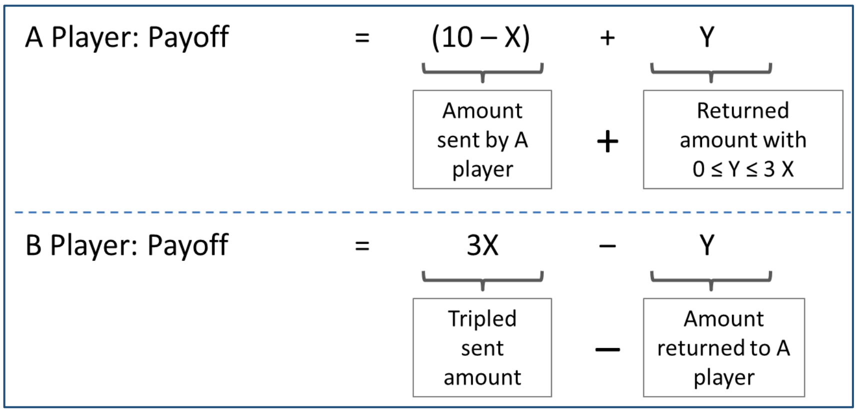

S5. Original Experimental Instructions (German)

VIELEN DANK FÜR IHRE TEILNAHME!
Bitte sprechen Sie nicht mit anderen TeilnehmerInnen während dem Experiment!

Liebe TeilnehmerInnen,

vielen Dank für Ihre Teilnahme an unserem Experiment. In diesem interessieren wir uns für Entscheidungen in Gruppen. Für den Erfolg unserer Studie ist es sehr wichtig, dass Sie sich die folgende Anleitung genau durchlesen.

Das Experiment wird ungefähr 2 Stunden Ihrer Zeit in Anspruch nehmen. Im Experiment können Sie Geld verdienen, wobei die Höhe Ihrer Auszahlung von Ihren Entscheidungen sowie den Entscheidungen anderer TeilnehmerInnen abhängt. Im Anschluss an das Experiment werden Sie einzeln und anonym in bar ausbezahlt. Während des Experiments sprechen wir nicht von Euro, sondern von Punkten. Die im Experiment erzielten Punkte werden bei der Auszahlung nach folgendem Wechselkurs umgerechnet:

10 Punkte = 1,00 Euro

Bitte nehmen Sie sich ausreichend Zeit beim Lesen der Anleitung und beim Treffen Ihrer Entscheidungen. Sie können durch eine schnelle Entscheidung die Dauer des Experimentes nicht beeinflussen. Bedenken Sie auch, dass die Höhe Ihrer Auszahlung von Ihren Entscheidungen abhängt. Vor Beginn des Experiments erhalten Sie einige Testfragen, mit deren Hilfe Sie überprüfen können, ob Sie die Anleitung verstanden haben. Für jede richtig beantwortete Testfrage erhalten Sie 1 Punkt. Zu keinem Zeitpunkt werden die Identität der TeilnehmerInnen und Ihre Auszahlungen aufgedeckt. Ihre Anonymität gegenüber den Studienleitern sowie den anderen TeilnehmerInnen bleibt gewahrt.

Wenn Sie beim Durchgehen der Anleitung Fragen haben, heben Sie bitte die Hand. Einer der Studienleiter wird dann zu Ihnen kommen und Ihre Frage privat beantworten. Bitte tauschen Sie sich nicht mit anderen TeilnehmerInnen aus und stellen Sie Ihre Fragen leise. Kommunikation zwischen den TeilnehmerInnen führt zum Ausschluss vom Experiment. Nach Beginn des Experiments dürfen keine weiteren Fragen gestellt werden.

Studienablauf

Zu Beginn des Experiments werden Sie zufällig in Gruppen von 9 TeilnehmerInnen aufgeteilt (Im Folgenden sprechen wir von Spielern). Jeder Spieler erhält eine eindeutige ID und eine von zwei möglichen Rollen zugeteilt: Rolle A oder Rolle B. Jede der neuner Gruppen setzt sich aus 3 Spielern mit der Rolle A (A-Spieler) und 6 Spielern mit der Rolle B (B-Spieler) zusammen. Die Gruppenzusammensetzung, die Rollenverteilung und die IDs der TeilnehmerInnen ändern sich während dem gesamten Ablauf des Experiments nicht: Sie interagieren mit denselben TeilnehmerInnen und behalten Ihre Rolle. Das Experiment dauert mindestens **24 Runden**, wobei es nach der 24. Runde in jeder weiteren Runde zufällig mit einer Wahrscheinlichkeit von 1/2 (=50%) endet. Jede der mindestens 24 identischen Runden besteht aus **5 Teilen**, die im Folgenden vorgestellt werden

1. Teil: In jeder Runde wird jedem der 3 A-Spieler zufällig genau ein B-Spieler zugeteilt, mit dem in der Runde interagiert wird. Zu Beginn der Runde erfährt der A-Spieler die Identität seines zugeordneten B-Spielers (B1, B2, B3, B4, B5 oder B6) und der B-Spieler die Identität des zugeordneten A-Spielers (A1, A2 oder A3). Die 3 nicht zugeordneten B-Spieler setzen für eine Runde aus. Anstatt mit einem A-Spieler zu interagieren werden den aussetzenden B-Spielern Rechenaufgaben gezeigt, die sie lösen können. Zum Lösen der Aufgaben haben die B-Spieler 30 Sekunden Zeit. Für jede gelöste Aufgabe gibt es zusätzlich 3 Punkte. Die folgenden Teile des Experiments beziehen sich nur auf A und B-Spieler, die miteinander interagieren.

2. Teil: Jeder A-Spieler erhält pro Runde eine Anfangsausstattung von 10 Punkten. Der A-Spieler hat im 2. Teil der Runde die Möglichkeit, dem B-Spieler einen Anteil seiner Anfangsausstattung zuzusenden. Dabei kann höchstens der gesamte Anfangsbetrag von 10 Punkten gesendet werden. Auf dem Weg zum B-Spieler wird der gesendete Betrag (im Folgenden X) verdreifacht (3X).

3. Teil: Im nächsten Schritt hat der B-Spieler die Möglichkeit, einen beliebigen Teil des verdreifachten gesendeten Betrags (3X) zurückzusenden. Der zurückgesendete Betrag (Im Folgenden Y) kann nicht größer als der verdreifachte gesendete Betrag sein ($0 \leq Y \leq 3X$).

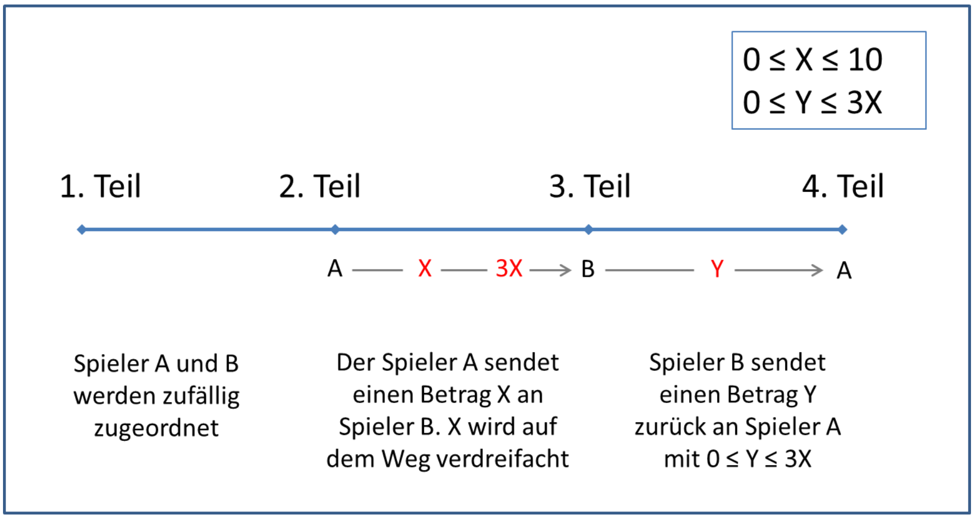

4. Teil: Der A-Spieler erfährt, welcher Betrag vom B-Spieler zurückgesendet wurde. Beide Spieler werden über das Geschehen in der Runde informiert und erfahren Ihre Auszahlung in Punkten aus der Runde sowie die Summe ihrer Auszahlungen über alle bisherigen Runden.

Die Auszahlungen der Spieler in einer Runde berechnen sich wie folgt:

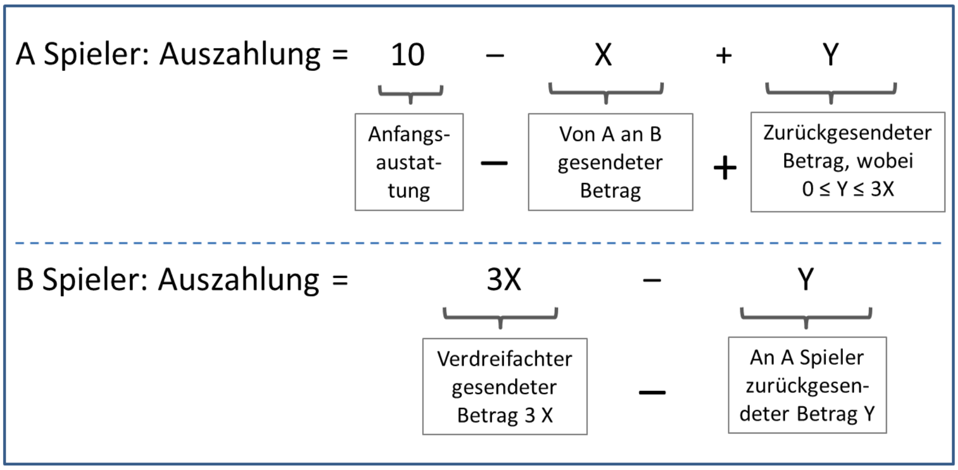

The following instructions are provided only in competition treatments

Rangliste der A-Spieler: Auf Basis der Summe der bisherigen Auszahlungen wird im 4. Teil eine Rangliste der 3 A-Spieler erstellt und den A-Spielern gezeigt. Der A-Spieler mit der höchsten Auszahlungssumme in Punkten über alle bisherigen Runden wird auf Rang 1 gesetzt. Der A-Spieler mit der zweithöchsten Summe auf Rang 2. Der A-Spieler mit der niedrigsten Summe auf Rang 3. Haben zwei A-Spieler die gleiche Punktzahl über alle Runden erworben, wird ihnen derselbe Rang zugewiesen. Nach jeweils 8 Runden (nach Ende der 8., der 16. und der 24. Runde) erhält der A-Spieler, der auf Rang 1 liegt, zusätzlich zu seinen bisherigen Auszahlungen einen Bonus von 100 Punkten (~10€). Der Spieler auf Rang 2 erhält einen Bonus von 50 Punkten (~5€). Der Spieler auf Rang 3 erhält keinen Bonus. Nach der 8., 16. und 24. Runde wird eine neue Rangliste erstellt, für die nur die Summe der Auszahlungen ab der 8. bzw. 16. Runde berücksichtigt werden (ohne die erworbenen Bonuszahlungen). Dem A-Spieler wird immer nur die Summe der Auszahlungen der bisherigen Runden ab der letzten Bonusauszahlung angezeigt. Für die B-Spieler gibt es keine Rangliste und keine Bonuszahlungen.

The following instructions are provided only in information sharing treatments

5. Teil: Zum Abschluss jeder Runde haben A-Spieler die Möglichkeit, ihre Erfahrungen aus der Runde mit anderen A-Spielern zu teilen. Hierbei können sich die A-Spieler entscheiden, ob sie mit keinem der beiden anderen A-Spieler, mit einem der beiden, oder mit beiden ihre Erfahrungen teilen möchten. Das Teilen von Informationen mit einem der beiden anderen A-Spieler kostet den mitteilenden A-Spieler einen Betrag C, der zwischen null Punkten und einem Punkt liegt ($0 \leq C \leq 1$). Der Betrag än-dert sich zufällig jede Runde und wird allen A-Spielern und B-Spielern zu Beginn der Runde mitgeteilt. Gibt ein A-Spieler Informationen an beide anderen A-Spieler weiter, so wird der zweifache Punktbe-trag (2C) von seiner Auszahlung in der Runde abgezogen. Wenn Informationen geteilt werden, so er-fahren der oder die ausgewählte(n) A-Spieler in der nächsten Runde die folgenden Informationen:

1. Mit welchem B-Spieler der Information teilende A-Spieler in der letzten Runde gespielt hat (B1-B6)
2. Welchen Betrag (X) der A-Spieler gesendet hat und die Höhe des Betrags nach Verdreifachung (3X)
3. Welchen Betrag (Y) der zugeordnete B-Spieler zurückgesendet hat.

Die von anderen A-Spielern zugespielten Informationen werden den A-Spielern in zwei Tabellen (eine Tabelle für jeden anderen A-Spieler) im 1. Teil der folgenden Runde angezeigt (siehe Screenshot nächste Seite). Jede Zeile der Tabellen stellt die geteilte Information aus einer Runde dar. Wurde kei-ne Information geteilt, so wird dies mit den Worten „Keine Info“ in der Tabelle angezeigt. Hinweis: Das Teilen von Informationen mit einem anderen A-Spieler heißt nicht, dass auch dieser A-Spieler mit Ihnen seine Informationen teilen möchte. Ein Informationsaustausch zwischen B-Spielern ist nicht möglich.

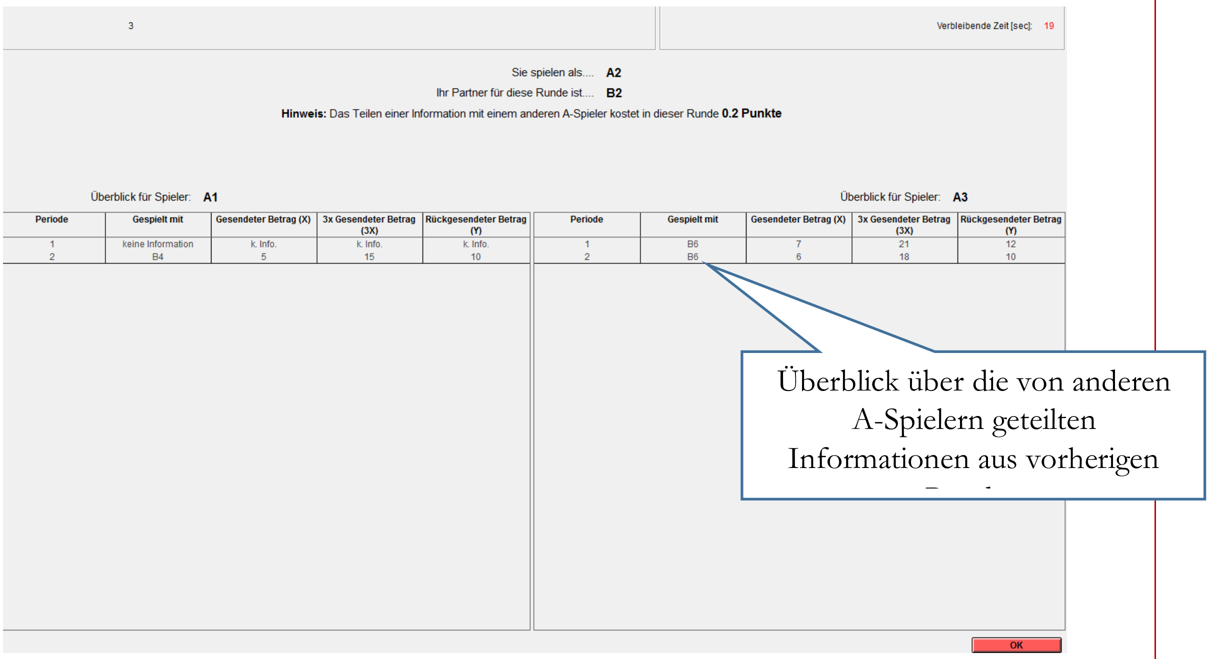

Im Anschluss an den 5. Teil startet die nächste von mindestens 24 Runden des Experiments. Während den einzelnen Runden des Experiments werden Sie wiederholt zu Ihren Erwartungen zum Verhalten Ihres Interaktionspartners befragt. Stimmen Ihre Erwartungen mit dem tatsächlich gezeigten Verhal-ten Ihrer Mitspieler überein, erhalten Sie eine zusätzliche Auszahlung am Ende des Experiments.

Nehmen Sie sich die Zeit, die Anleitung erneut genau durchzulesen. Wenn Sie eine Frage haben soll-ten, heben Sie bitte die Hand. Auf der folgenden Seite finden Sie einen Überblick zur Anleitung. Bitte lassen Sie diesen immer aufgedeckt an Ihrem Platz liegen.

Überblick Anleitung

Bitte immer aufgedeckt haben

Insgesamt 9 Spieler pro Gruppe, 3 A-Spieler und 6 B-Spieler

Mindestens 24 Runden. Jede Runde besteht aus 5 Teilen

Nach der 24. Runde endet das Experiment in jeder Runde mit Wahrscheinlichkeit 50%

**1. Teil**	- A und B-Spieler werden zufällig zugeordnet und erfahren, mit wem sie in der Runde interagieren<br>- A-Spieler erhalten Informationen, die von anderen A-Spielern geteilt wurden (sharing treatment)<br>- Nicht zugeordnete B-Spieler lösen Rechenaufgaben und erhalten 3 Punkte pro richtiger Lösung
**2. Teil**	- A-Spieler erhalten pro Runde 10 Punkte als Anfangsausstattung<br>- A-Spieler können Betrag X an zugeordneten B-Spieler senden ($0 \leq X \leq 10$).<br>- Der Betrag X wird auf dem Weg zu B verdreifacht (3X)
**3. Teil**	- B-Spieler können Betrag Y zurück an A-Spieler senden. Y nicht größer als 3X ($0 \leq Y \leq 3X$)
**4. Teil**	- A und B-Spieler erfahren Spielverlauf und ihre Auszahlungen sowie die Summe der Auszahlungen über alle bisherigen Runden<br>- A-Spieler erfahren, auf welcher Rangposition sie und die anderen A-Spieler sich befinden.<br>- Der Rang hängt von der Summe der Auszahlungen aus den bisherigen Runden ab<br>- Nach der 8., 16. und 24. Runde startet die Rangliste neu und es gibt eine Bonusauszahlung von 100 Punkten für Erstplatzierte bzw. 50 Punkten für Zweitplatzierte (only competition treatment)
**5. Teil**	- A-Spieler können ihre Erfahrungen aus der Runde mit anderen A-Spielern teilen (sharing treatment)<br>- Das Teilen von Informationen kostet einen Betrag C, der jede Runde zwischen 0 und 1 variiert<br>-Die ausgewählten A-Spieler erfahren, mit welchem B-Spieler in der letzten Runde interagiert wurde, welcher Betrag (X) gesendet wurde, und wie viel zurückgesendet wurde (Y)

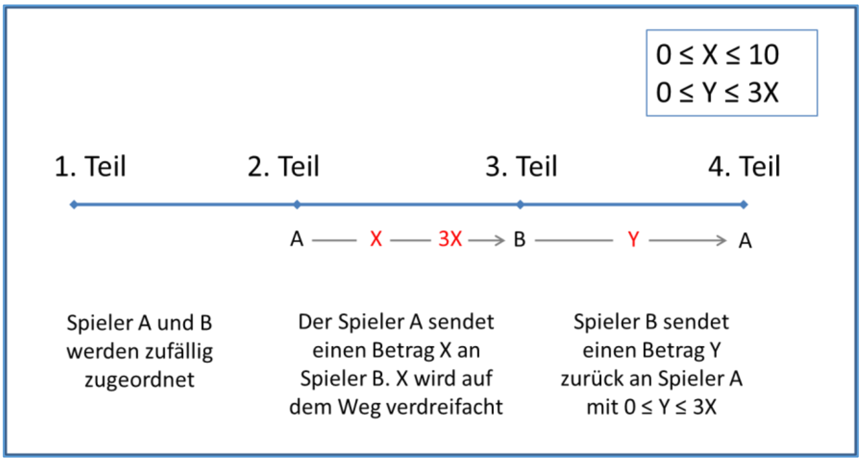

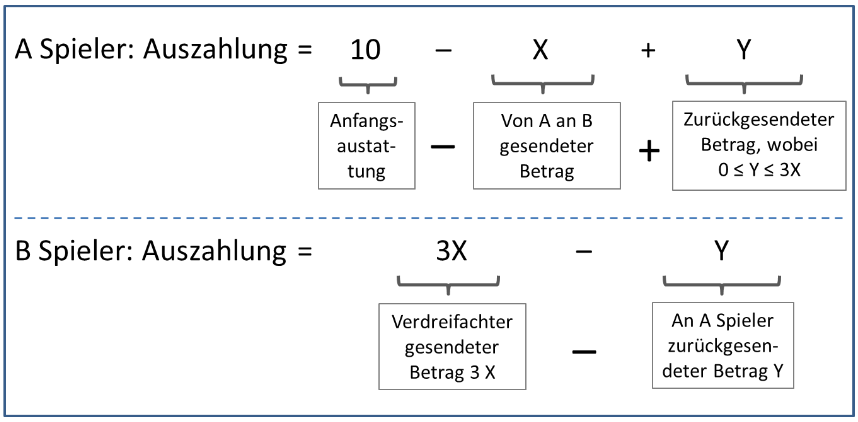

S6. Test Questions for Participants

Please answer the following questions. You earn 1 token for each correctly answered question.

Question	Answer
1. At the beginning of the experiment all participants are assigned to groups.	
a) How many A-players does each group consist of?	3
b) How many rounds are at least played in the experiment?	24
c) Does the player composition of the groups change throughout the experiment?	No
2. You are playing as an A-player. In this round you send 0 tokens to your assigned B-player.	
a) What is your payoff in this round?	10
b) What is the payoff of the B-player in this round?	0
3 You are playing as a B-player. You were randomly matched with an A-player in this round. The A-player sends you 8 tokens. The sent amount (X) is increased to 24 token. From these 24 tokens you return 16 tokens to the A-player.	
a) By which factor is the sent amount multiplied before reaching the B-player?	3
b) What is the payoff of the A-player in this round?	18
c) What is your payoff in this round?	8
d) What would be the payoff of the A-player if you would send back nothing?	2
e) What would be your payoff if you would send back nothing?	24

S7. Screenshots from Experiment

Trustor screen: sending decision

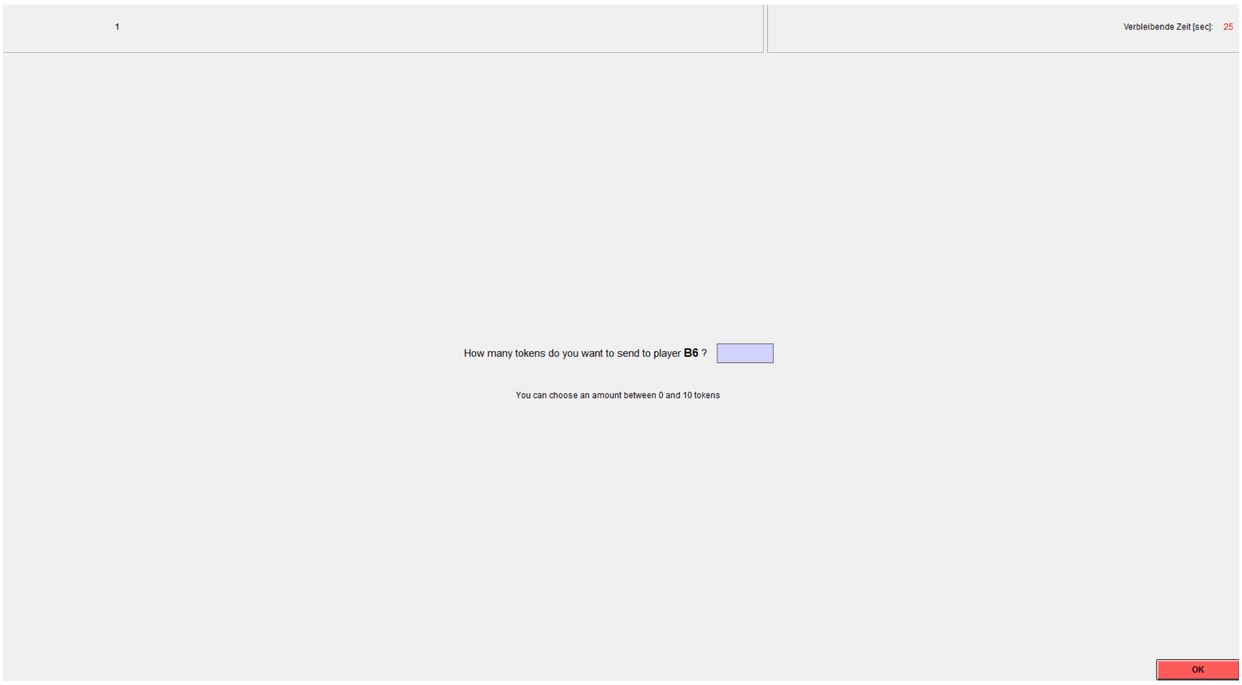

Trustee screen: resending decision

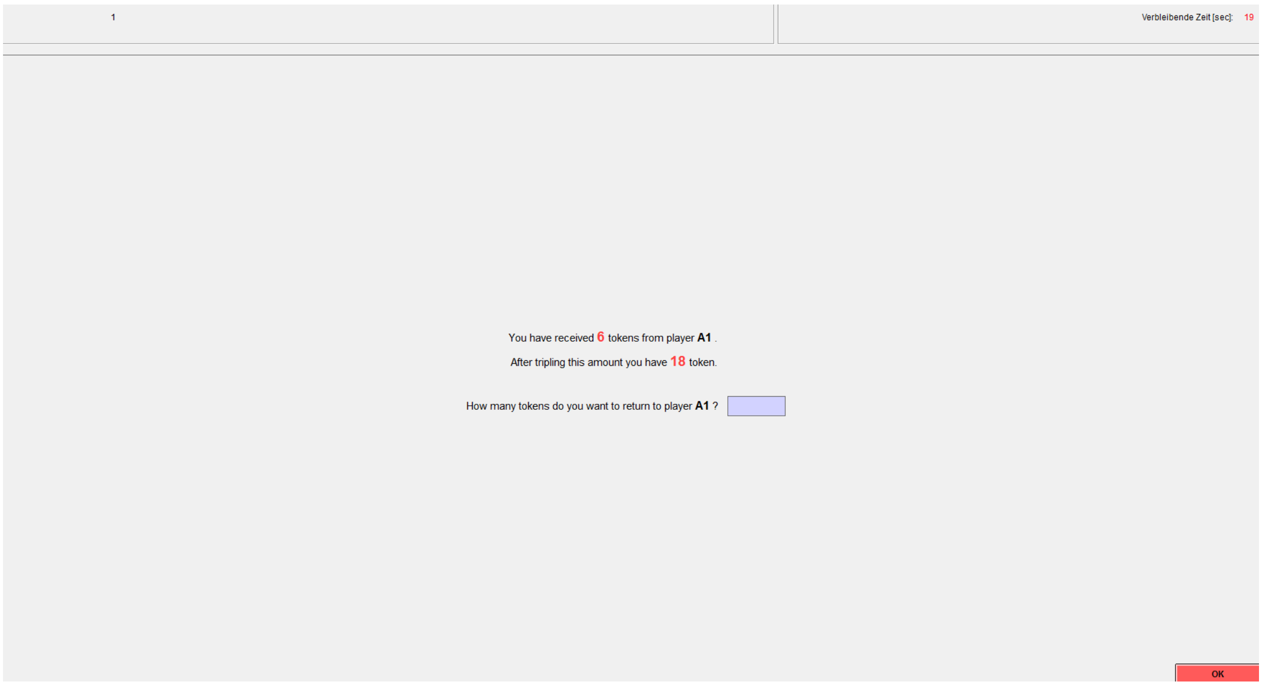

Trustor screen: beliefs about resending behavior

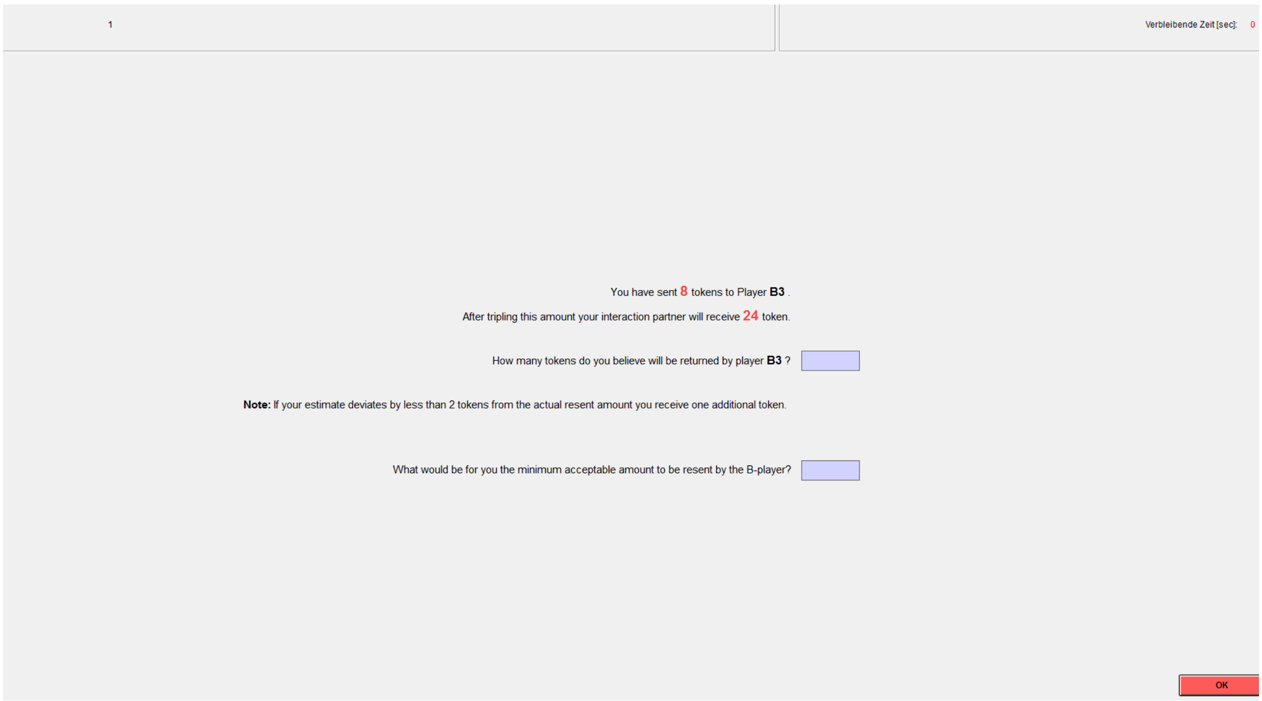

Trustor screen: payoffs and tournament ranking

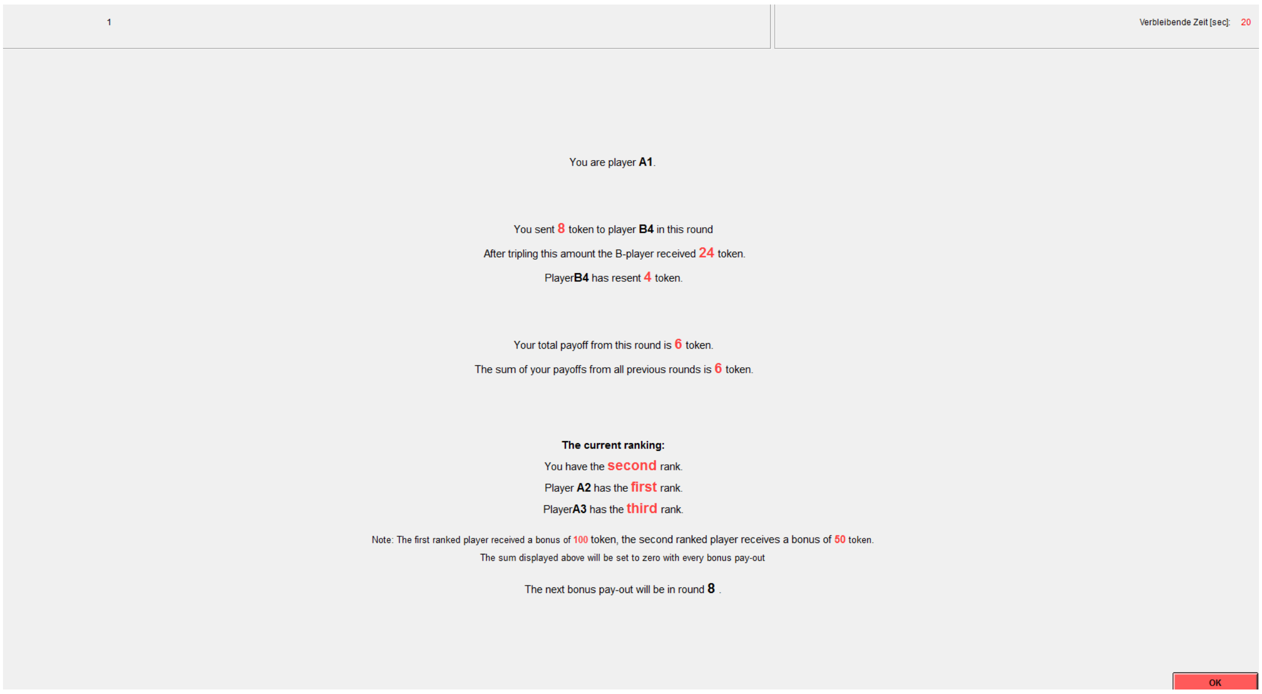

Trustor screen: information sharing decision

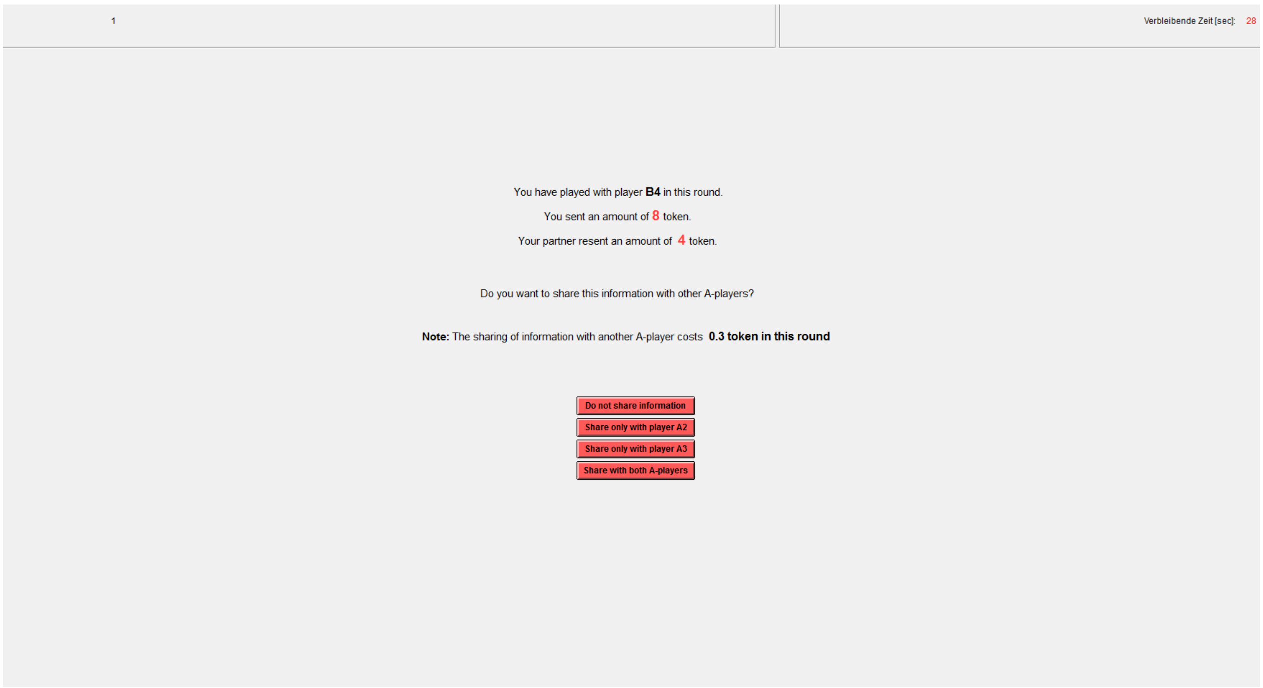

Trustee screen: beliefs about information sharing behavior

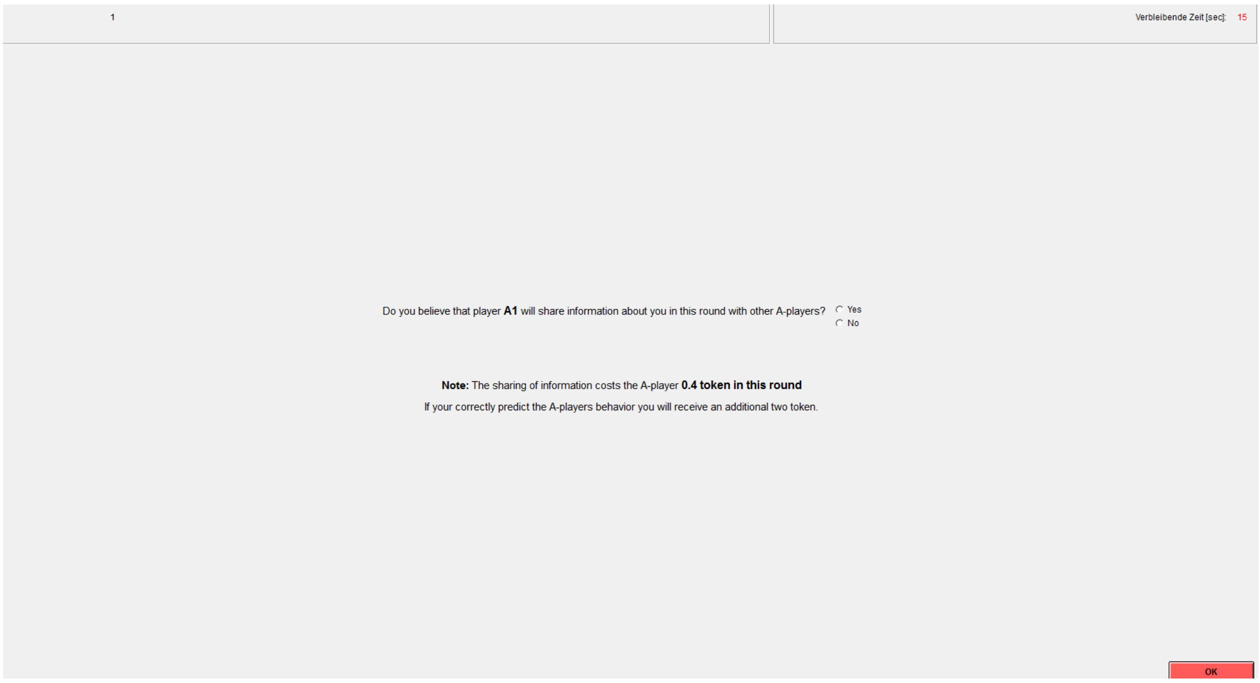

References

Abimbola, T. & Vallaster, C., 2007. Brand, organisational identity and reputation in SMEs: An overview. *Qualitative Market Research: An International Journal*, 10(4), pp.341–348.

Akerlof, G.A., 1970. The Market for "Lemons": Quality Uncertainty and the Market Mechanism. *The Quarterly Journal of Economics*, 84(3), p.488.

Anderhub, V., Engelmann, D. & Güth, W., 2002. An experimental study of the repeated trust game with incomplete information. *Journal of Economic Behavior & Organization*, 48(2), pp.197–216.

Banerjee, A., Breza, E., Chandrasekhar, A.G., Duflo, E. & Jackson, M.O., 2012. Come Play with Me: Experimental Evidence of Information Diffusion About Rival Goods. *Mimeo*.

Bohnet, I., Harmgart, H., Huck, S. & Tyran, J.-R., 2005. Learning trust. *Journal of the European Economic Association*, 3(May 2005), pp.322–329.

Bohnet, I. & Huck, S., 2004. Repetition and reputation: Implications for trust and trustworthiness when institutions change. *American Economic Review*, 94(2), pp.362–366.

Bolton, G., Greiner, B. & Ockenfels, A., 2013. Engineering Trust: Reciprocity in the Production of Reputation Information. *Management Science*, 59(2), pp.265–285.

Bolton, G.E., Katok, E. & Ockenfels, A., 2004. How Effective Are Electronic Reputation Mechanisms? An Experimental Investigation. *Management Science*, 50(11), pp.1587–1602.

Bolton, G.E., Ockenfels, A. & Ebeling, F., 2011. Information value and externalities in reputation building. *International Journal of Industrial Organization*, 29(1), pp.23–33.

Brown, M., Falk, A. & Fehr, E., 2004. Relational contracts and the nature of market interactions. *Econometrica*, 72(3), pp.747–780.

Brown, M. & Zehnder, C., 2005. Credit Registries , Relationship Banking and Loan Repayment. *Science*, 39(240).

Brown, M. & Zehnder, C., 2007. Credit reporting, relationship banking, and loan repayment. *Journal of Money, Credit and Banking*, 39(8), pp.1883–1918.

Cabrales, A., Charness, G. & Villeval, M.C., 2011. Hidden information, bargaining power, and efficiency: An experiment. *Experimental Economics*, 14(2), pp.133–159.

Camerer, C. & Thaler, R.H., 1995. Anomalies: Ultimatums, Dictators and Manners. *Journal of Economic Perspectives*, 9(2), pp.209–219.

Casari, M., 2007. Emergence of endogenous legal institutions: Property rights and community governance in the Italian Alps. *Journal of Economic History*, 67(1), pp.191–226.

Chaudhuri, A., 2011. Sustaining cooperation in laboratory public goods experiments: A selective survey of the literature. *Experimental Economics*, 14(1), pp.47–83.

Clay, K., 1997. Trade without law: Private-order institutions in Mexican California. *Journal of Law, Economics, and Organization*, 13(1), pp.202–231.

Deephouse, D.L., 2000. Media Reputation as a Strategic Resource: An Integration of Mass Communication and Resource-Based Theories. *Journal of Management*, 26(6), pp.1091–1112.

Diekmann, A., Jann, B., Przepiorka, W. & Wehrli, S., 2014. Reputation Formation and the Evolution of Cooperation in Anonymous Online Markets. *American Sociological Review*, 79(1), pp.65–85.

Falk, A. & Fischbacher, U., 2006. A theory of reciprocity. *Games and Economic Behavior*, 54(2), pp.293–315.

Fehr, E. & Gächter, S., 2002. Altruistic punishment in humans. *Nature*, 415(6868), pp.137–140.

Fehr, E. & Gächter, S., 1998. Reciprocity and economics: The economic implications of Homo Reciprocans. *European Economic Review*, 42(3–5), pp.845–859.

Fombrun, C. & Shanley, M., 1990. What's in a Name? Reputation Building and Corporate Strategy. *Academy of Management Journal*, 33(2), pp.233–258.

Fombrun, C.J., 1996. Reputation: Realizing Value from the Corporate Image, Boston: Harvard Business Press.

Gërxhani, K., Brandts, J. & Schram, A., 2013. The emergence of employer information networks in an experimental labor market. *Social Networks*, 35(4), pp.541–560.

Greif, A., 2006. Institutions and the path to the modern economy: Lessons from medieval trade, Cambridge: Cambridge University Press.

Güth, W., 1995. On ultimatum bargaining experiments - A personal review. *Journal of Economic Behavior and Organization*, 27(3), pp.329–344.

Güth, W., Schmittberger, R. & Schwarze, B., 1982. An experimental analysis of ultimatum bargaining. *Journal of Economic Behavior and Organization*, 3(4), pp.367–388.

Hoffman, P.T., Postel-Vinay, G. & Rosenthal, J.-L., 1999. Information and economic history: how the credit market of Old Regime Paris forces us to rethink the transition to capitalism. *American historical review*, 104, pp.69–94.

Houser, D. & Wooders, J., 2006. Reputation in auctions: Theory, and evidence from eBay. *Journal of Economics and Management Strategy*, 15(2), pp.353–369.

Huck, S., Lünser, G.K. & Tyran, J.R., 2012. Competition fosters trust. *Games and Economic Behavior*, 76(1), pp.195–209.

Keser, C., 2002. Trust and reputation building in E-Commerce. *Recherche*, 22533, pp.1–36.

Kreps, D.M. & Wilson, R., 1982. Reputation and imperfect information. *Journal of Economic Theory*, 27(2), pp.253–279.

Milgrom, P. & Roberts, J., 1982. Predation, reputation, and entry deterrence. *Journal of Economic Theory*, 27(2), pp.280–312.

Millon, M.H. & Thakor, A. V, 1985. Moral Hazard and Information Sharing: A Model of Financial Information Gathering Agencies. *Journal of Finance*, 40(5), pp.1403–1422.

Padilla, A.J. & Pagano, M., 2000. Sharing default information as a borrower discipline device. *European Economic Review*, 44(10), pp.1951–1980.

Pagano, M. & Jappelli, T., 1993. Information Sharing in Credit Markets. *The Journal of Finance*, 48(5), pp.1693–1718.

Resnick, P., Zeckhauser, R., Swanson, J. & Lockwood, K., 2006. The value of reputation on eBay: A controlled experiment. *Experimental Economics*, 9(2), pp.79–101.

Rindova, V.P., Williamson, I.O., Petkova, A.P. & Sever, J.M., 2005. Being good or being known: An empirical examination of the dimensions, antecedents, and consequences of organizational reputation. *Academy of Management Journal*, 48(6), pp.1033–1049.

Schelling, T.C., 1960. The Strategy of Conflict, Cambridge: Harvard University Press.

Turban, D.B. & Greening, D.W., 1997. Corporate Social Performance and Organizational Attractiveness to Prospective Employees. *Academy of Management Journal*, 40(3), pp.658–672.
